# Antioxidant Aryl-Substituted Phthalan Derivatives Produced by Endophytic Fungus *Cytospora rhizophorae*

**DOI:** 10.3389/fchem.2022.826615

**Published:** 2022-02-14

**Authors:** Hongxin Liu, Zhaoming Liu, Yanjiang Zhang, Yuchan Chen, Huan Wang, Haibo Tan, Weimin Zhang

**Affiliations:** ^1^ State Key Laboratory of Applied Microbiology Southern China, Guangdong Provincial Key Laboratory of Microbial Culture Collection and Application, Guangdong Open Laboratory of Applied Microbiology, Institute of Microbiology, Guangdong Academy of Sciences, Guangzhou, China; ^2^ National Engineering Research Center of Navel Orange, Gannan Normal University, Ganzhou, China; ^3^ Key Laboratory of South China Agricultural Plant Molecular Analysis and Genetic Improvement, Guangdong Provincial Key Laboratory of Applied Botany, South China Botanical Garden, Chinese Academy of Sciences, Guangzhou, China

**Keywords:** *Cytospora rhizophorae*, endophytic fungus, antioxidtant activity, *Gynochthodes officinalis*, cytorhizophin

## Abstract

Six new phthalan derivatives cytorhizophins D-I (1-6) as well as three known derivatives cytorhizophin C, pestacin and rhizophol B were isolated from *Cytospora rhizophorae.* Among them, cytorhizophins D-E (1-2) and F-G (3-4) were two pairs of diastereoisomers, all of them featuring a 1-phenyl-1,3-dihydroisobenzofuran scaffold with a highly oxygenated *O*-linked isopentenyl unit. Besides, cytorhizophins H-I (5-6) represent the first examples of phthalide family with fascinating 6/6/6/5 tetracyclic ring system fusing as unprecedented furo [4,3,2-*kl*]xanthen-2 (10b*H*)-one skeleton. The structures of the new phthalan derivatives were extensively confirmed by detail spectroscopic analysis. The partial absolute configurations of compounds 1-6 were established through electronic circular dichroism (ECD) calculations. Moreover, compounds 1-4 showed remarkable antioxidant activities with EC_50_ values ranging from 5.86 to 26.80 μM, which were better than or comparable to that of ascorbic acid (positive control).

## Introduction

The free radicals and reactive oxygen species (ROS) were highly reactive intermediates widely existing in human body, which can react with human biomolecules including lipids, proteins, DNA, etc, thus causing seriously detrimental health effects, such as neurodegenerative diseases, atherosclerosis, liver cirrhosis, cataracts, diabetes, and cancer (Kang et al., 2007; López-Alarcónand Denicola, 2013). With the aim to clear up the oxidative stress resulting by excess amounts of ROS, numerous remarkable results have been reported in the past decades (Cerutti, 1985; Halliwell, 1987; Breimer, 1990; Ding et al., 1999; Grisham et al., 2000; Aitken et al., 2012; Russell and Cotter, 2015; El-Hawary et al., 2019; Kusio et al., 2020). Among them, antioxidant was respected as one of the most efficient therapeutic strategies against human diseases related to oxidative damage by ROS (Beckman et al., 1992; Taniguchi et al., 1993).

In the repertoire of pharmaceutical antioxidant discovery and achievements, natural products exampled by astaxanthin, vitamins, as well as carotenoids have played extremely significant roles (Quiñones, et al., 2012). Additionally, many attentions have been continuously paid to the discovery of natural antioxidants. Consequently, more and more nature-originated antioxidants were emerged and widely used in functional foods, pharmaceutical drugs, and industrial cosmetics (Mussard et al., 2019; Wen et al., 2017). Polyphenols represent a characteristic family of natural-based organic compounds with strong antioxidant activities (Dao et al., 2020; Bodoira and Maestri, 2020). Phthalans featured by a core isobenzofuran skeleton were a typical class of phenols, which have dramatically attracted many medicinal scientists attributable to their affluent structure diversities, novel architecture complexities, and significant pharmaceutical activities in recent years (Naito and Kaneko, 1969; Strobel et al., 2002; Harper et al., 2003; Kapoor et al., 2003; Fotso et al., 2008). Especially, the 1-phenyl-phthalan moiety is frequently encountered in numerous natural products and commercially available drugs or drug lead compounds. Their fascinating biological activities and novel structural features rendered them appealing targets for the natural product and pharmaceutical communities.

**FIGURE 1:**
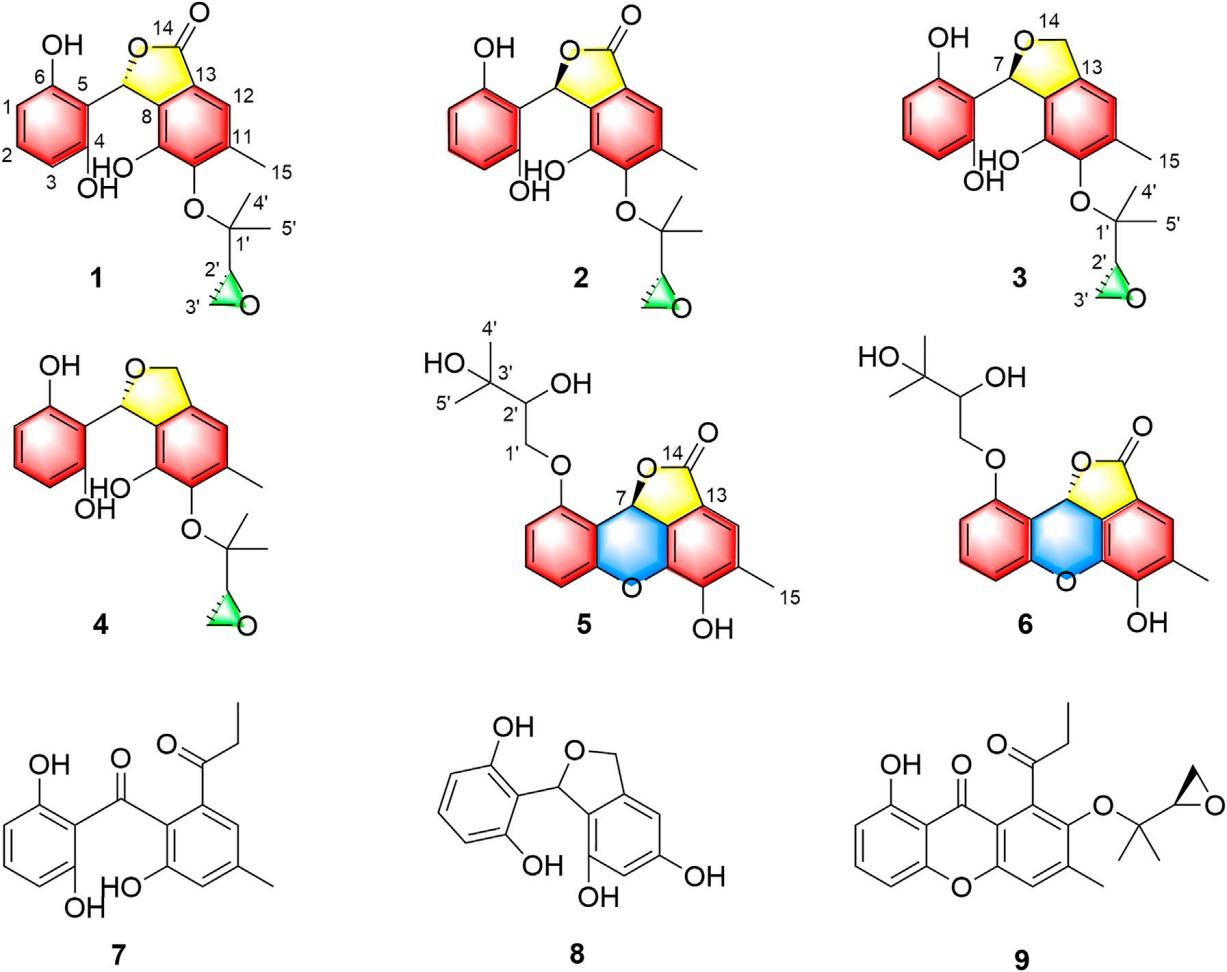
Structures of compounds **1-9**.

As a part of our continuing program to discover structurally unique natural products with significantly biological potentials from the endophytic fungi (Liu et al., 2017; Liu et al., 2019a; Liu H.-X. et al., 2019; Chen et al., 2019), an endophytic fungus, *Cytospora rhizophorae* A761, was obtained from the stem of *Gynochthodes officinalis* (F.C.How) Razafim. and B. Bremer (basionym: *Morinda officinalis*). The chemical investigation on the liquid culture of *C. rhizophorae* has resulted in the successful purification of six novel polyphenolic natural products cytorhizophins D-I (**1**-**6**) as well as three known derivatives cytorhizophin C (**7**) (Liu et al., 2019c), pestacin (**8**) (Harper et al., 2003) and rhizophol B (**9**) (Liu et al., 2019c) (Figure 1). Cytorhizophins D-E (**1**-**2**) and F-G (**3**-**4**) were two pairs of diastereoisomers, all of them featured a 1-phenyl-1,3-dihydroisobenzofuran scaffold with a highly oxygenated isopentenyl unit. Cytorhizophins H-I (**5**-**6**) represent the first examples of phthalide family with a fascinating 6/6/6/5 tetracyclic ring system fusing as unprecedented furo [4,3,2-*kl*]xanthen-2 (10b*H*)-one skeleton. Herein, the details of the extraction, purification, structure elucidation, and antioxidant activity of cytorhizophins D-I were described.

## Materials and Methods

### General Experimental Procedures

The general experimental procedures were described in supporting information.

### Fungal Material

The information of fungal material used in this study were identical to that of the previous descriptions (Liu et al., 2019a).

### Extraction and Isolation

The strain *Cytospora rhizophorae* A761 was kept for 7 days at 28°C and 120 r/m on a rotary shaker in 150 flasks (1,000 ml) containing 500 ml of potato dextrose broth (potato 20%, glucose 2%, K_2_HPO_4_ 0.3%, MgSO_4_•7H_2_O 0.15%, vitamin B 10 mg/L). The fermented broth (75 L) was filtered through cheesecloth to give the broth and mycelia. The fermented broth were subjected to macroporous resin D101 column with ethanol as eluent. The EtOH fraction was concentrated under a vacuum to yield a dark brown gum (37 g). The crude extract was subjected to reversed-phase silica gel C_18_ using step gradient elution with MeOH/H_2_O, 60%→100% to afford six fractions (Fr.): Fr.1-Fr.6.

Then, Fr. 2 (8.92 g) was separated by silica gel flash CC (*n*-hexane/EtOAc, 20:1→1:1, v/v) to give nine subfractions (Fr.2-1 to Fr.2-9). Fr.2-5 (166.2 mg) was subjected to CC on Sephadex LH-20 (CH_2_Cl_2_/MeOH, 1:1, v/v) to give four sub-fractions (Fr. 2-5-1 to Fr. 2-5-4). Fr. 2-5-4 was further purified by silica gel flash column chromatography (*n*-hexane/EtOAc, 10:1→1:1, v/v) to give **8** (9.0 mg). Fr.2-7 (3.07 g) was subjected to CC on Sephadex LH-20 (CH_2_Cl_2_/MeOH, 1:1, v/v) to give ten sub-fractions (Fr. 2-7-1 to Fr. 2-7-10). Fr. 2-7-5 was purified by silica gel flash column chromatography and further purified by semipreparative HPLC (MeOH/H_2_O, 60:40, v/v, 3 ml/min) to give **9** (5.0 mg). Fr. 2-7-6 was divided into five sub-fractions (Fr.2-7-6-1 to Fr. 2-7-6-5) by silica gel flash column chromatography (*n*-hexane/EtOAc, 10:1→1:1, v/v). Fr. 2-7-6-5 was further separated by semipreparative HPLC (MeOH/H_2_O, 73:27, v/v, 3 ml/min) to give four sub-fractions (Fr. 2-7-6-5-1 to Fr. 2-7-6-5-4). Fr. 2-7-6-5-2 (10 mg, *t*_R_ = 8.3 min) was purified by semipreparative HPLC equipped with a Chiralpak IC column (*n*-hexane 95%/isopropyl alcohol, 7:3, 3 ml/min) to obtain **3** (5.0 mg, *t*_R_ = 8.6 min) and **4** (2.5 mg, *t*_R_ = 9.0 min).

Fr. Three was further purified by CC over reversed-phase silica gel C_18_ (MeOH/H_2_O, 20%→100%) to give five subfractions (Fr.3-1 to Fr.3-5). Fr.3-2 (2.0 g) was divided into seven sub-fractions (Fr. 3-2-1 to Fr. 3-2-7) by Sephadex LH-20 (CH_2_Cl_2_/MeOH, 1:1, v/v). Fr. 3-2-1 was further purified by repeated silica gel and semi-preparative HPLC (ACN/H_2_O, 50:50, v/v, 3 ml/min) to obtain compound **7** (2.0 mg, *t*_R_ = 12.0 min). Fr. 3-2-2 was subjected by silica gel CC (*n*-hexane/EtOAc, 5:1→1:2, v/v) to yield four sub-fractions (Fr.3-2-2-1 to Fr. 3-2-2-4). Fr. 3-2-2-1 was purified by semipreparative HPLC (MeOH/H_2_O, 60:40, v/v, 3 ml/min) to give a mixture (10 mg, *t*_R_ = 8.9 min). The mixture was further separated by HPLC (Chiralpak IC column, *n*-hexane 95%/isopropyl alcohol, 4:1, 3 ml/min) to obtain **2** (4.0 mg, *t*_R_ = 20.8 min) and **1** (4.0 mg, *t*_R_ = 25.1 min).

Fr.3-4 (1.3 g) was separated by silica gel flash CC (*n*-hexane/EtOAc, 5:1→1:5, v/v) to yield twelve sub-fractions (Fr.3-3-1 to Fr. 3-3-12). Compound **5** (3.0 mg, *t*_R_ = 14.0 min) was obtained from Fr. 3-3-10 by semipreparative HPLC (MeOH/H_2_O, 60:40, v/v, 3 ml/min). Fr.3-4 (2.8 g) was separated by Sephadex LH-20 (CH_2_Cl_2_/MeOH, 1:1, v/v) to give seven sub-fractions (Fr. 3-4-1 to Fr. 3-4-7). Fr. 3-4-4 was divided into four sub-fractions (Fr.3-4-4-1 to Fr. 3-4-4-4) by silica gel flash CC (*n*-hexane/EtOAc, 2:1→1:2, v/v). Fr. 3-4-4-2 was purified by HPLC (ACN/H_2_O, 55:45, v/v, 2 ml/min) to yield compound **6** (4.0 mg, *t*_R_ = 18.7 min).

Cytorhizophin D (**1**): yellow powder [*α*]^25^_D_ = +34.0 (*c* 0.12, MeOH); CD (MeOH, 0.4 mg/ml): 206 (−5.3), 214 (+40.5), 230 (+8.6), 247 (−18.2), 260 (+1.9), 288 (−2.3), 306 (−1.7) nm; UV (MeOH) *λ*_max_ (log *ε*) 213 (5.35), 311 (4.23) nm; IR *ν*_max_ 3,230, 2,927, 1716, 1,616, 1,472, 1,015, 887, 794 cm^−1^. For ^1^H and ^13^C NMR, see Table 1; HRESIMS: *m*/*z* 373.1285 [M + H]^+^ (calcd for C_20_H_21_O_7_, 373.1282).

**TABLE 1:** ^1^H (600 MHz) and^13^C (150 MHz) NMR data of **1** and **2** in CD_3_COCD_3_.

No	1		2	
	*δ*_H_	*δ*_C_	*δ*_H_	*δ*_C_
1	6.38, d, 8.1	106.9, CH^a^	6.38, d, 8.1	106.8, CH^a^
2	6.98, t, 8.1	130.4, C	6.99, t, 8.1	130.4, C
3	6.38, d, 8.1	106.9, CH^a^	6.38, d, 8.1	106.8, CH^a^
4		157.9, C		157.9, C
5		108.4, C		108.3, C
6		157.9 C		157.9, C
7	7.12, s	75.7, CH	7.16, s	75.7, CH
8		111.9, C		112.0, C
9		152.0, C		151.9, C
10		141.2, C		141.0, C
11		142.7, C		142.7, C
12	6.74, s	117.4, CH	6.74, s	117.3, CH
13		145.1, C		145.3, C
14		171.7, C		171.1, C
15	2.31, s	17.9, CH_3_	2.28, s	17.9, CH_3_
1′		82.2, C		81.6, C
2′	3.13, dd, 4.2, 2.7	56.8, CH	3.17, dd, 4.2, 2.7	56.5, CH
3a′	2.69, m	43.9, CH_2_	2.61, m	43.9, CH_2_
3b′	2.55, dd, 4.2, 2.7		2.51, dd, 4.2, 2.7	
4′	1.20, s	23.7, CH_3_	1.22, s	22.1, CH_3_
5	1.14, s	21.0, CH_3_	1.17, s	21.9, CH_3_

a Detected by HMBC.

Cytorhizophin E (**2**): yellow powder [*α*]^25^_D_ = −22.3 (*c* 0.02, MeOH); CD (MeOH, 0.3 mg/ml): 206 (+3.8), 214 (−33.5), 231 (−5.3), 247 (+12.9), 262 (−2.3), 288 (+0.9), 308 (+0.6) nm; UV (MeOH) *λ*_max_ (log *ε*) 213 (5.33), 310 (4.26) nm; IR *ν*_max_ 3,236, 2,926, 1715, 1,614, 1,470, 1,427, 1,268, 1,233, 1,198, 1,162, 1,017, 977, 903, 885, 795, 752, 739 cm^−1^. For ^1^H and ^13^C NMR, see Table 1; HRESIMS: *m*/*z* 373.1281 [M + H]^+^ (calcd for C_20_H_21_O_7_; 373.1282).

Cytorhizophin F (**3**): yellow powder [*α*]^25^_D_ = +127 (*c* 0.02, MeOH); CD (MeOH, 0.15 mg/ml): 207 (+108.0), 238 (−2.4), 286 (−5.2) nm; UV (MeOH) *λ*_max_ (log *ε*) 284 (4.15) nm; IR *ν*_max_ 3,415, 2,953, 1,616, 1,597, 1,472, 1,285, 1,225, 1,140, 1,225, 1,015, 926, 887, 864, 826, 795, 753, 738 cm^−1^. For ^1^H and ^13^C NMR, see Table 2; HRESIMS: *m*/*z* 359.1497 [M + H]^+^ (calcd for C_20_H_23_O_6_, 359.1489).

**TABLE 2:** ^1^H (600 MHz) and^13^C (150 MHz) NMR data of **3** and **4** in CD_3_COCD_3_.

No	3		4	
	*δ*_H_	*δ*_C_	*δ*_H_	*δ*_C_
1	6.40, d, 8.1	108.0, CH	6.40, d, 8.1	108.0, CH
2	6.96, t. 8.1	129.0, CH	6.96, t. 8.1	129.0, CH
3	6.40, d, 8.1	108.0, CH	6.40, d, 8.1	108.0, CH
4		156.0, C		156.0, C
5		112.2, C		112.2, C
6		156.0, C		156.0, C
7	6.78, br s	79.3 CH	6.79, br s	79.3 CH
8		125.0, C		125.0, C
9		147.5, C		147.5, C
10		140.6, C		140.6, C
11		133.9, C		133.9, C
12	6.51, s	117.4, CH	6.51, s	117.4, CH
13		135.4, C		135.4, C
14	5.52, dd, 12.2, 2.4	72.3, CH_2_	5.49, dd, 12.2, 2.3	72.3, CH_2_
14	5.10, dd, 12.2, 2.4		5.15, dd, 12.2, 2.3	
15	2.21, s	16.8, CH_3_	2.21, s	16.7, CH_3_
1′		80.3, C		80.3, C
2′	3.16, dd, 4.5, 2.7	57.2, CH	3.17, dd, 4.5, 2.7	57.3, CH
3a′	2.75, t, 4.5	44.0, CH_2_	2.76, t, 4.5	44.0, CH_2_
3b′	2.70, dd, 4.5, 2.7		2.69, dd, 4.5, 2.7	
4′	1.27, s	23.3, CH_3_	1.27, s	23.2, CH_3_
5′	1.19, s	21.7, CH_3_	1.18, s	21.6, CH_3_

Cytorhizophin G (**4**): yellow powder [*α*]^25^_D_ = ‒76 (*c* 0.08, MeOH); CD (MeOH, 0.10 mg/ml): 207 (‒75.3), 238 (+2.7), 287 (+4.7) nm; UV (MeOH) *λ*_max_ (log *ε*) 282 (4.07) nm; IR *ν*_max_ 3,290, 1,616, 1,474, 1,472, 1,225, 1,015, 887, 795 cm^−1^. For ^1^H and ^13^C NMR, see Table 2; HRESIMS: *m*/*z* 359.1493 [M + H]^+^ (calcd for C_20_H_23_O_6_, 359.1489).

Cytorhizophin H (**5**): pale yellow powder [*α*]^25^_D_ = ‒117.5 (*c* 0.06, MeOH); CD (MeOH, 0.2 mg/ml): 202 (+42.2), 214 (−33.5), 235 (−4.2), 243 (−8.7) nm; UV (MeOH) *λ*_max_ (log *ε*) 311 (4.01), 282 (3.90) nm; IR *ν*_max_ 3,303, 2,828, 1715, 1,462, 1,423, 1,281, 1,236, 1,192, 1,144, 1,045, 1,011, 902, 786 cm^−1^. For ^1^H and ^13^C NMR, see Table 3; HRESIMS: *m*/*z* 373.1285 [M + H]^+^ (calcd for C_20_H_21_O_7_, 373.1282).

**TABLE 3:** ^1^H (600 MHz) and^13^C (150 MHz) NMR data of **5** and **6** in CD_3_COCD_3_.

No	5		6	
	*δ*_H_	*δ*_C_	*δ*_H_	*δ*_C_
1	6.76, d, 8.1	106.3, CH	6.62, d, 8.1	111.8, CH
2	7.07, t, 8.1	129.4, C	7.10, t, 8.1	130.1, C
3	6.38, d, 8.1	106.3, CH	6.47, d, 8.1	115.3, CH
4		156.5, C		156.5, C
5		113.1, C		111.6, C
6		158.3, C		160.5, C
7	7.28, s	76.0, CH	7.04, s	75.6, CH
8		141.0, C		141.5, C
9		144.8, C		143.5, C
10		151.2, C		151.3, C
11		142.5, C		142.6, C
12	6.71, s	118.2, CH	6.75, s	118.0, CH
13		111.0, C		111.5, C
14		171.3, C		171.3, C
15	2.24, s	19.0, CH_3_	2.27, s	18.0, CH_3_
1′a	4.70, dd, 12.4, 4.3	70.0, CH_2_	4.52, d, 11.8	79.9, CH_2_
1′b	4.26, dd, 12.4, 8.9		4.33, dd, 11.8, 5.0	
2′	3.89, dd, 8.8, 4.3	72.9, CH	4.16, t, 5.0	79.1, CH
3′		86.0, C		84.3, C
4′	1.44, s	28.3, CH_3_	1.35, s	21.5, CH_3_
5′	1.38, s	18.5, CH_3_	1.25, s	22.5, CH_3_

Cytorhizophin I (**6**): pale yellow powder [*α*]^25^_D_ = ‒39.3 (*c* 0.02, MeOH); CD (MeOH, 0.2 mg/ml): 200 (−38.5), 214 (−30.2), 237 (−3.3), 243 (+7.4) nm; UV (MeOH) *λ*_max_ (log *ε*) 310 (3.87), 284 (3.84) nm; IR *ν*_max_ 3,302, 1,472, 1,238, 1,016, 903, 677, 600, 592, 556 cm^−1^. For ^1^H and ^13^C NMR, see Table 3; HRESIMS: *m*/*z* 373.1284 [M + H]^+^ (calcd for C_20_H_21_O_7_, 373.1282).

### DPPH Photometric Assay

The DPPH photometric assay were carried out according to our previously established method (Zhong et al., 2020).

## Results and Discussion

Cytorhizophin D (**1**) was purified as a yellow powder, and the molecular formula of **1** had been established as C_20_H_20_O_7_ by HRESIMS with an obvious ion peak discovered at *m*/*z* 373.1285 ([M + H]^+^, calcd for C_20_H_21_O_7_, 373.1282). The IR spectrum of **1** displayed prominent resonance bands at 3,230 and 1,716 cm^−1^, clarifying the existence of hydroxy and carbonyl functionality. The ^1^H NMR spectrum of **1** exhibited four downshifted protons at *δ*_H_ 6.74 (1H, s, H-12), *δ*_H_ 6.98 (1H, t, *J* = 8.2 Hz, H-2), 6.38 (1H, d, *J* = 8.2 Hz, H-1), and 6.38 (1H, d, *J* = 8.2 Hz, H-3), which were responsive for two independent benzenoid rings. Moreover, the signals for three methyl groups [*δ*_H_ 1.14 (3H, s), 1.20 (3H, s), 2.31 (3H, s)] signals were also observed in its ^1^H NMR spectrum. Additionally, the ^13^C NMR data (Table 1) coupling with HSQC data of **1** further identified 20 carbon signals, which could be readily differentiated to three methyls, two methylenes, six methines, as well as nine quaternary carbons with a carbonyl moiety (*δ*_C_ 171.7).

**FIGURE 2:**
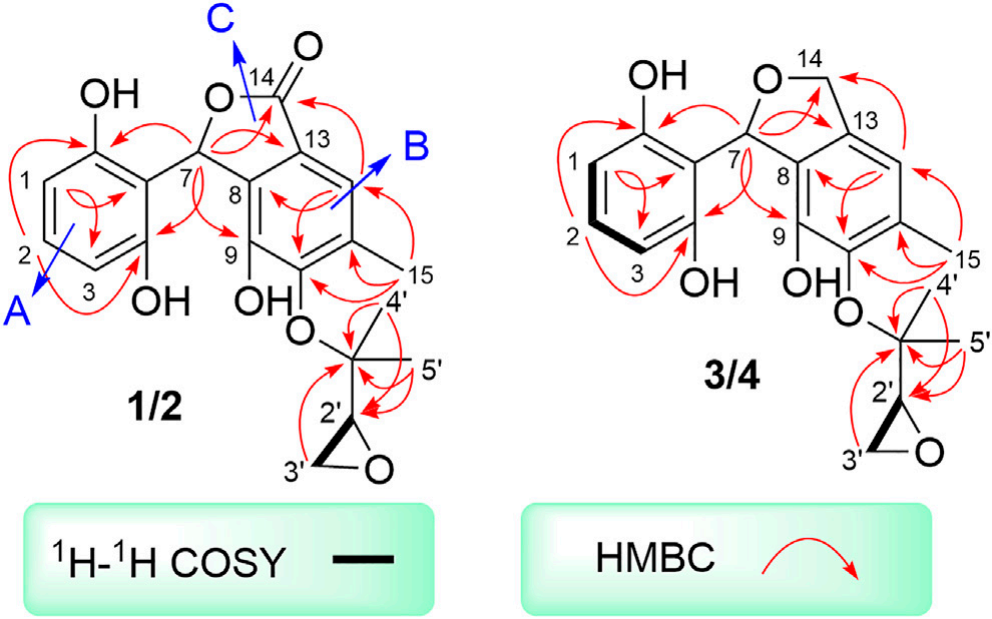
^1^H-^1^H COSYs and key HMBCs of **1-4**.

Two spin systems of C-1/C-2/C-3 and C-2′/C-3′ were successfully assigned by carefully analysis of the ^1^H-^1^H COSY spectrum of **1** (Figure 2). As referring to the fragment C-1/C-2/C-3, the critical HMBC correlative signals from H-1 to C-3 and C-5, H-2 to C-4 and C-6 in conjunction with consideration of the overlapping NMR data of C-1/C-3 as well as C-2/C-4 confirmed the presence of a symmetric aromatic ring. Moreover, the conclusive HMBC correlative signals from H_3_-15 to C-10, C-11, and C-12 as well as H-12 to C-8, C-10 and C-14 revealed the existence of the ring B. The linkage of rings A and B *via* C-7 methine was successfully verified by the unambiguous HMBC correlations from H-7 to C-4, C-6, C-9, and C-13. The five-membered lactone ring C was then established with the aid of the conclusive HMBC correlations from the critical proton H-7 to C-13 and C-14. Moreover, the key HMBC correlations from H-7 to C-9, and H-12 to C-14 concluded that the lactone ring C was fused with the benzene ring B to construct the key phthalide core.

Furthermore, with the aid of the spin fragment C-2′/C-3′, a highly oxygenated isopentyl unit was strongly suggested to connect with the phthalide core through an ether bond in **1**, answering for the informative HMBC correlations from the methyl protons H_3_-4′ to C-1′, C-2′ together with H_3_-5′ to C-1′, C-2′. Moreover, there was an epoxy ring in the isopentyl unit, which could be further concluded through the high-field shift of C-2′ (*δ*_C_ 56.8) and C-3′ (*δ*_C_ 43.9) together with the molecular formula. Because the lack of direct HMBC correlations from the isopentyl unit and the ring B, the location of isopentyl moiety could be readily assigned at C-10 position by comparing the carbon resonance shifts of the C-9 (*δ*_C_ 152.0) and C-11 (*δ*_C_ 142.7) along with the definitive NOESY correlations from methyls H_3_-4′ and H_3_-5′ to H_3_-15. Consequentially, the planar structure of **1** was finally elucidated as outlined in Figure 1.

Cytorhizophin E (**2**) was obtained as a yellow powder and found to possess a molecular formula of C_20_H_20_O_7_ based on the HRESIMS ion peak at *m*/*z* 373.1281 [M + H]^+^, indicating eleven indices of hydrogen deficiency. The IR spectrum of **2** was quite similar to that of **1**. The inspection of the NMR data (Table 1) of **2** with those of **1** demonstrated that **2** displayed close similarity with **1**. The obvious differences were the chemical shifts of the H-7 (*δ*_H_ 7.16 ppm for **1** versus 7.12 ppm for **2**), H-15 (*δ*_H_ 2.31 ppm for **1** versus 2.28 ppm for **2**), H-2′ (*δ*_H_ 3.13 ppm for **1** versus 3.17 ppm for **2**), H-3′ (*δ*_H_ 2.69, 2.55 ppm for **1** versus 2.61, 2.51 ppm for **2**), H_3_-4′ (*δ*_H_ 1.20 ppm for **1** versus 1.22 ppm for **2**), and H_3_-5′ (*δ*_H_ 1.14 ppm for **1** versus 1.17 ppm for **2**), which strongly concluded that the compounds **1** and **2** should be a pair of diastereoisomers.

**FIGURE 3:**
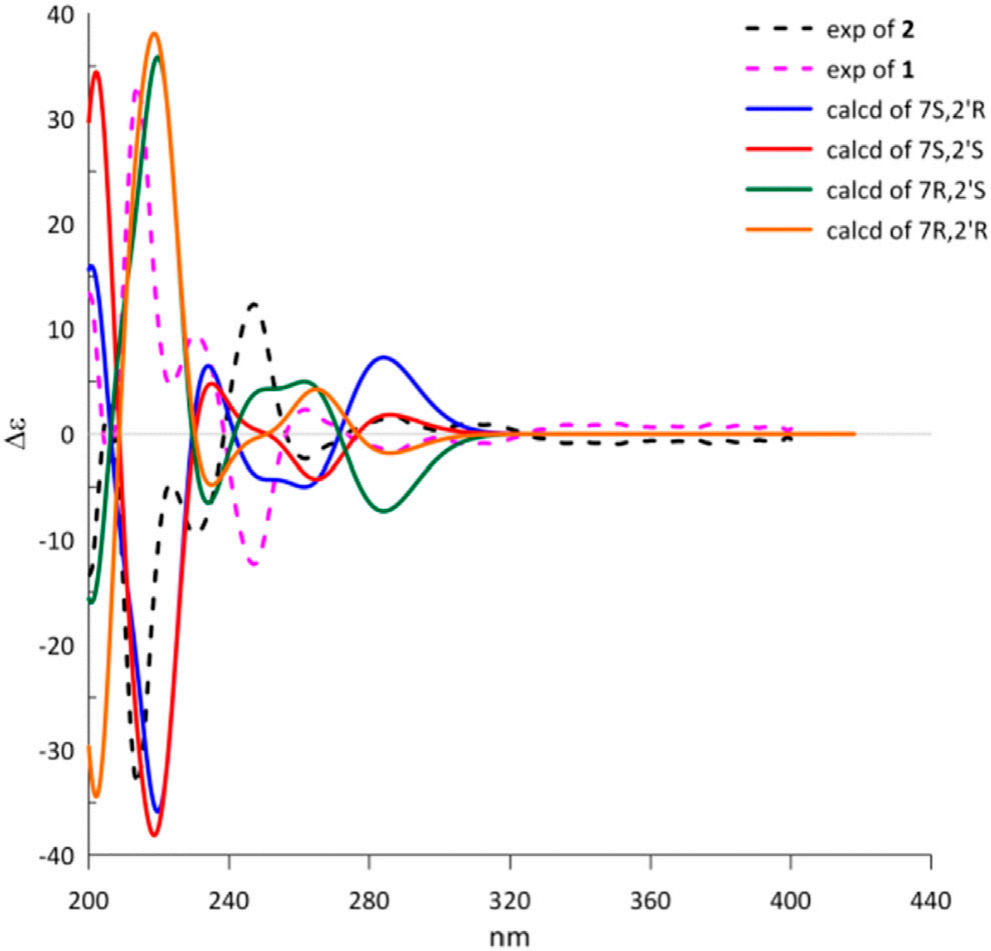
Experimental and calculated ECD spectra of **1** and **2**.

To determine the absolute configuration of **1** and **2**, the experimental and TDDFT calculated circular dichroism (CD) spectra at cam-b3lyp/def2svp level were performed. The calculated ECD spectrum with 7*R* configuration showed very excellent similarities to those of the experimental CD spectrum of **1** as shown in Figure 3. Thus, the absolute configuration at C-7 of **1** was rationally assigned as *R*. Moreover, the calculated ECD Cotton effects of the 7*S* enantiomer were well agreement with those in the experimental ECD spectrum of **2**. Cytorhizophins D (**1**) and E (**2**) possessed a couple of distant stereogenic centers at C-7 and C-2′ positions, which made the establishment for the absolute configuration of C-2′ to be challenged. The absolute configuration of C-2′ was deducted as *R* as that of the co-isolated compound rhizophol B, which was confirmed by X-ray diffraction (Liu Z. et al., 2019). Therefore, the absolute stereochemistries for **1** and **2** were clarified as 7*R*,2′*R* and 7*S*,2′*R*.

Cytorhizophin F (**3**) was also afforded as a yellow powder. The molecular formula of **3** was confirmed as C_20_H_22_O_6_ by its (+)-HRESIMS *m*/*z* 359.1497 [M + H]^+^. The 1D NMR spectroscopic data of the natural product **3** showed a collection of typical resonance signals responsive for a 1,2,3-trisubstituted benzene ring and an isopentyl unit, which showed very close similarity to the structure of **1**. After a detail inspection and interpretation of 1D NMR spectra of **3**, it could readily disclose that its planar structure should be closely similar to that of **1**, and the major difference between them was the absence of carbonyl group in compound **3**. This conclusion further strengthened on the basis of the signals for the *O*-substituted methylene [*δ*_C_ 72.3, *δ*_H_ 5.10 (dd, *J* = 2.4, 12.2 Hz), 5.22 (dd, *J* = 2.4, 12.2 Hz)] in **3** instead of a carbonyl functionality in **1**. Moreover, the informative HMBC correlative signals from H-7 to C-4, C-6, C-9, C-13, and C-14 could further strengthen this deduction. Thus, the planar structure of **3** was completely elucidated as depicted in Figure 1.

**FIGURE 4:**
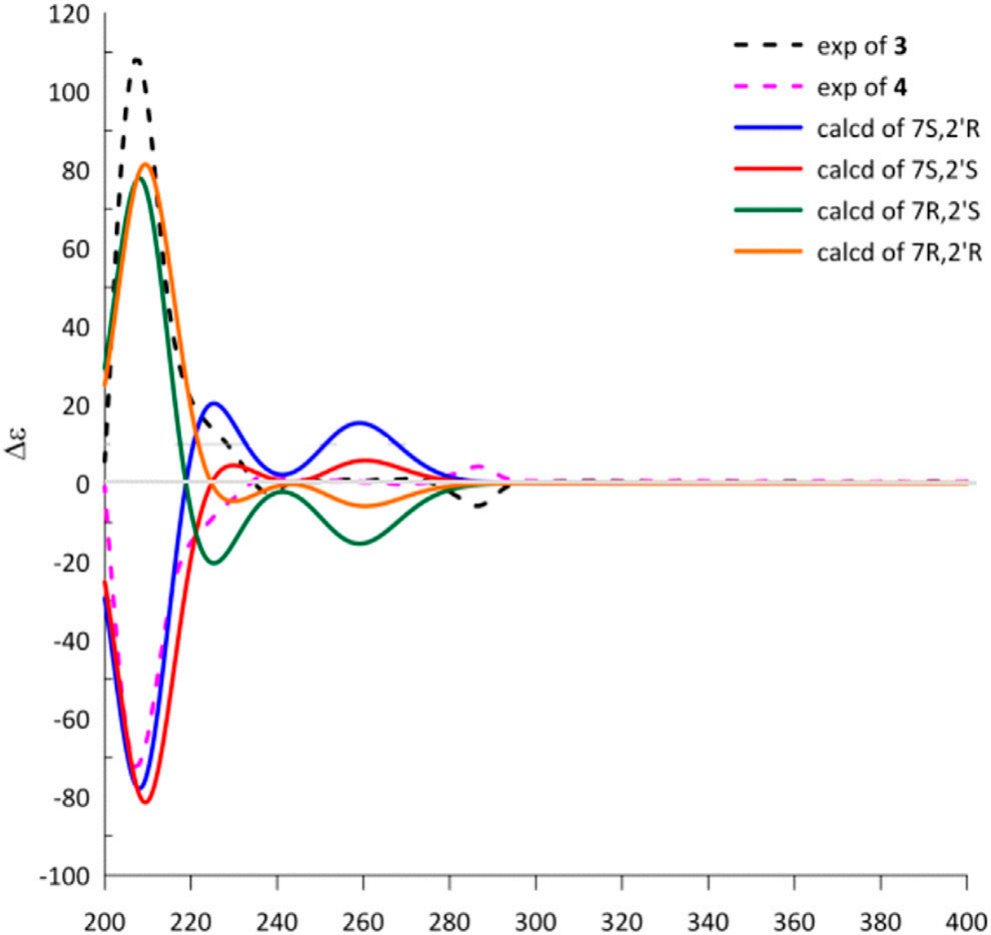
Experimental and calculated ECD spectra of **3** and **4**.

Cytorhizophin G (**4**) was obtained to be a yellow powder and had same molecular formula with that of **3** as determined by its HREIMS ion peak at *m*/*z* 359.1493 [M + H]^+^, revealing ten degrees of unsaturation. Obviously, the ^13^C NMR spectroscopic data and HSQC spectrum of **4** collectively suggested 20 carbon signals, and all of them showed very similar chemical shifts to those of **3**. Their little differences between chemical shifts implied that they shared the same planar structure. For compound **4**, the Cotton effects in the ECD spectrum were almost direct contrary to those of **3**, suggesting that **4** should share the opposite configuration at C-7 position comparing with **3**, attributed to the slight contribution of C-2′ chiral center. The configuration of chiral center for C-7 was further confirmed by ECD calculations in Figure 4. The results showed that the theoretical ECD curve for 7*R* agreed with the experimental plot of **3**, 7*S* was matched with the experimental plot of **4**. Therefore, the absolute configurations of **3** and **4** were successfully established as 7*R*,2′*R*, 7*S*,2′*R*, correspondingly.

**FIGURE 5:**
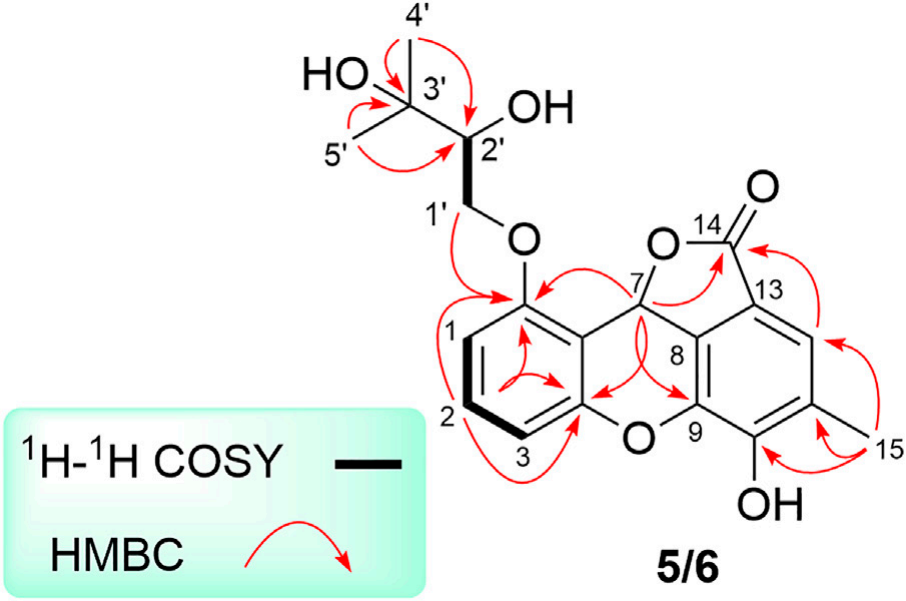
Key ^1^H-^1^H COSY, and HMBC correlations of **5** and **6**.

Cytorhizophin H (**5**) was isolated as a pale yellow powder and assigned an HRESIMS ion peak at *m*/*z* 373.1285 [M + H]^+^ (C_20_H_21_O_7_, calcd 373.1282), which perfectly agreed the molecular formula of C_20_H_20_O_7_ and showed 11 degrees of hydrogen deficiency. The ^1^H NMR spectrum of **5** exhibited four aromatic protons [*δ*_H_ 7.28 (1H, s, H-12), 7.07 (1H, t, *J* = 8.1 Hz, H-2), 6.76 (1H, d, *J* = 8.1 Hz, H-1), 6.38 (1H, d, *J* = 8.1 Hz, H-3)] as well as the characteristic proton resonance signals of three methyls [*δ*_H_ 2.24 (3H, s, H_3_-15), 1.44 (3H, s, H_3_-4′), 1.38 (3H, s, H_3_-5′)].

Analysis of 1D as well as 2D NMR spectra including COSY, HSQC, and HMBC could readily finish the preliminary construction of the planar structure of **5** as shown in Figure 5. Firstly, the obvious HMBC correlations from H-1 to C-3 and C-5, H-2 to C-4 and C-6 along with the pivotal spin system C-1/C-2/C-3 successfully evidenced the presence of a 1,2,3-trisubstituted aromatic ring A. Secondly, as referring to the other spin system of C-1′/C-2′, the existence of a highly oxygenated C-5 isopentyl unit was then verified with the aid of the HMBC correlative signals from H_3_-4′ to C-2′ and C-3′, H_3_-5′ to C-2′ and C-3′. Moreover, the location of isopentyl functionality had been assigned to attach at C-6 position in the ring A through the C-1′-O-C-6 ether bond, attributable to the decisive HMBC cross-peak from H-1′ to C-6. Additionally, the resulting penta-substituted ring B was finally established and clarified by the HMBC correlations from H-12 to C-8, C-10 and C-14, H_3_-15 to C-10, C-11, and C-12. Taking the degrees of unsaturation into account, the assignment of a benzopyran ring between C-4 and C-9 *via* a fused oxygen bridge was eventually verified. Moreover, the asymmetrical ^1^H and ^13^C NMR signals for the typical trisubstituted aromatic ring C further strengthened the conclusion. Therefore, the planar structure of **5** was determined and established to possess a fascinating 6/6/6/5 tetracyclic ring system fusing as unusual furo [4,3,2-*kl*]xanthen-2 (10bH)-one skeleton and showed in Figure 1.

Cytorhizophin I (**6**) was separated as a pale yellow powder and assigned an HRESIMS ion peak at *m*/*z* 373.1285 [M + H]^+^ (calcd for C_20_H_21_O_7_, 373.1282), which perfectly agreed with the molecular formula of C_20_H_20_O_7_ and showed 11 degrees of hydrogen deficiency. The ^1^H NMR spectrum of **6** exhibited four aromatic protons [*δ*_H_ 6.75 (1H, s, H-10), 7.10 (1H, t, *J* = 8.1 Hz, H-2), 6.62 (1H, d, *J* = 8.1 Hz, H-1), and 6.47 (1H, d, *J* = 8.1 Hz, H-3)], suggesting the existence of two phenyl rings. Interestingly, close comparison of the NMR data of compounds **5** and **6** as shown in Table 3 indicated that these two compounds ought to share a significantly similar core structure. The further HMBC correlations analysis collectively pointed to that the compounds **5** and **6** were a pair of diastereoisomers.

**FIGURE 6:**
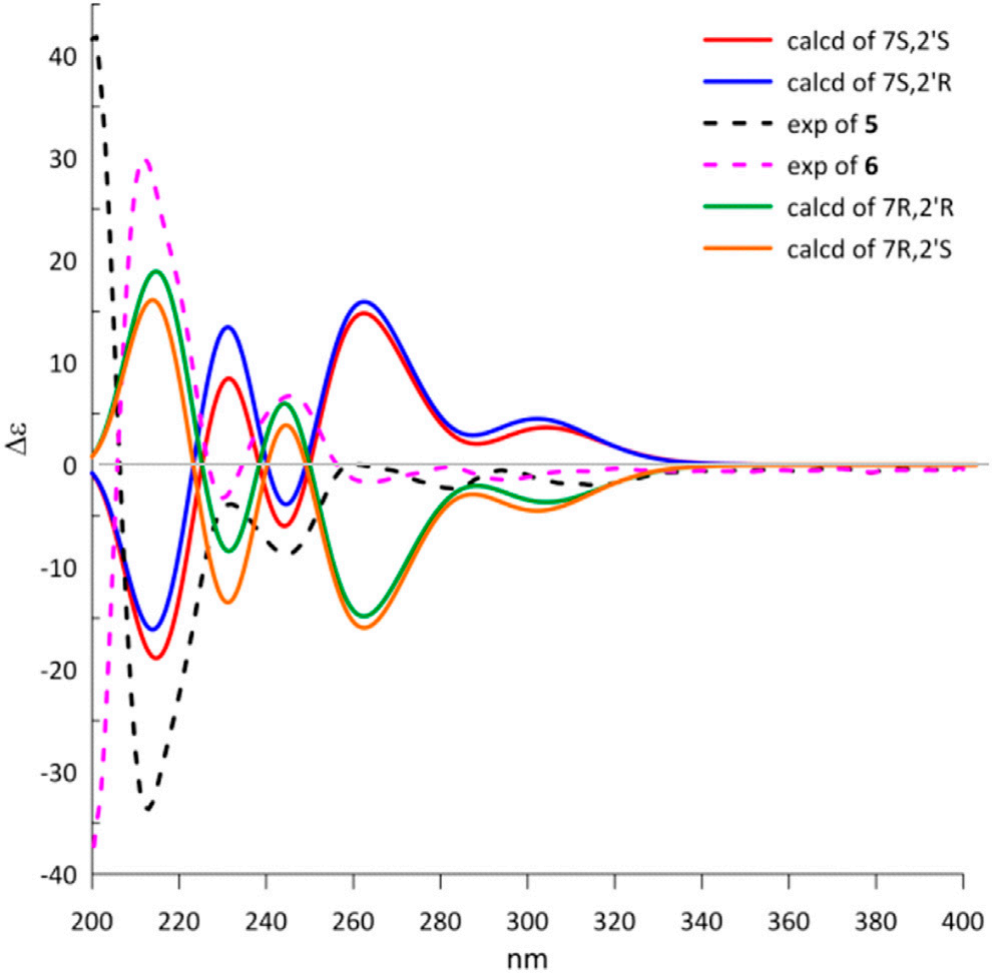
Experimental and calculated ECD spectra of **5** and **6**.

The natural products **5** and **6** possessed two distant stereocenters C-7 and C-2′. In order to establish their absolute configurations of diastereoisomers **5** and **6**, the effort towards theoretical ECD calculation at b3lyp/6-311 + *g* (d,p) level were performed. The results revealed that the theoretical ECD plots of 7*S* and 7*R* matched with the experimental spectra of **5** and **6**, respectively, which allowed to establish the absolute configurations of 7*S* for **5** and 7*R* for **6** (Figure 6). Unsatisfactorily, the absolute configuration for C-2′ was failed to be assigned by the little amount and the lack of CD contribution.

**SCHEME 1:**
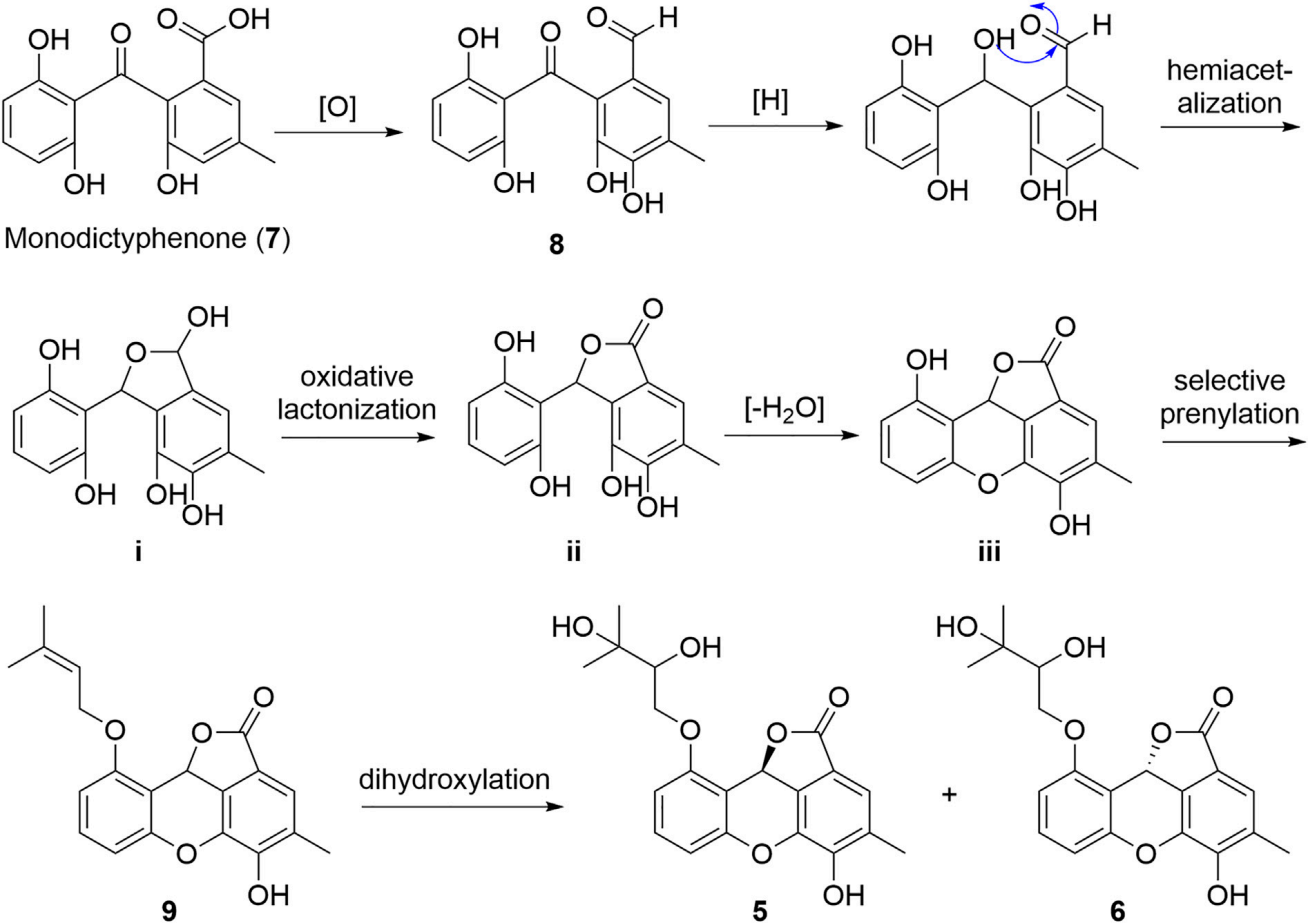
Plausible Biosynthetic Pathways of **5** and **6**.

Due to the structural novelty cytorhizophins H-I (**5**-**6**) with fascinating 6/6/6/5 tetracyclic furo [4,3,2-kl]xanthen-2 (10bH)-one skeletons, their biogenetic pathways were proposed as shown in Scheme 1. Cytorhizophins H-I (**5**-**6**) were bio-originated from the monodictyphenone (**7**), the following selective oxidation, reduction, and hemiacetalization transformations would result the critical intermediate **i**, which underwent selective oxidative lactonization and dehydrated to generate the key precursors **ii** and **iii**, respectively. Then, the selective prenylation of **iii** further gave rise to cytorhizophins H-I (**5**-**6**).

The characteristic of polyhydroxy groups in these new compounds logically suggested that they might possess antioxidant activity. The further experimental testing confirmed that compounds **1**-**6** indeed showed significant antioxidant activity as evaluated by DPPH (2,2-diphenyl-1-picrylhydrazyl) scavenging assay and described in the Experimental part (Coteele et al., 1996; Mensor et al., 2001). Compounds **1-4** showed remarkable DPPH radical scavenging activities with EC_50_ values ranging from 5.86 to 26.80 μM, which are better than that of the positive control ascorbic acid (EC_50_ of 25.53 μM). Compounds **5** and **6** were found to be weak DPPH scavengers at a concentration of 100 μM (Table 4). From a comparison of the structures of compounds **1**-**4** with compounds **5** and **6**, it could be readily found that the opening of the middle ring might play the predominant roles in enhancing their DPPH scavenging capacity.

**TABLE 4:** Antioxidant activities of compounds **1**-**6**.

Compounds	EC_50_ (μM)^a^
	DPPH radical scavenging
1	17.39 ± 0.94
2	26.80 ± 0.62
3	5.86 ± 0.71
4	7.72 ± 0.36
5	>100
6	>100
Ascorbic acid	25.53 ± 0.21

a EC_50_ is defined as the concentration sufficient to obtain 50% of a maximum effect estimate in 100%, Values are expressed as the mean ± SD.

## Conclusion

The chemical research on the endophytic fungus *Cytospora rhizophorae* has disclosed a new range of antioxidative ingredients*,* involving six novel phthalan derivatives named as cytorhizophins D-I (**1**-**6**). Among them, cytorhizophins D-E (**1**-**2**) and F-G (**3**-**4**) were two pairs of diastereoisomers, and all of them featuring a 1-phenyl-1,3-dihydroisobenzofuran scaffold with a highly oxygenated *O*-linked isopentenyl unit; whereas cytorhizophins H-I (**5**-**6**) represent the first examples of phthalide family with a fascinating 6/6/6/5 tetracyclic ring system fusing as unprecedented furo [4,3,2-*kl*]xanthen-2 (10b*H*)-one skeleton. Compounds **1**-**4** showed significant DPPH radical scavenging activities with EC_50_ values ranging from 5.86 to 26.80 μM, which are much better than that of the positive control ascorbic acid (EC_50_ of 25.53 μM). Therefore, the preliminary results revealed that cytorhizophins D-G might be served as promising lead compounds for the development of bio-available potent anti-oxidant drugs. The detailed potential mechanisms to explain the antioxidant action of these compounds is now underway and will be reported in due course.

## Data Availability

The datasets presented in this study can be found in online repositories. The names of the repository/repositories and accession number(s) can be found below: https://www.ncbi.nlm.nih.gov/genbank/, KU529867.
