# Supplementary material for: Antioxidant Aryl-Substituted Phthalan Derivatives Produced by Endophytic Fungus Cytospora rhizophorae
Source: Front Chem. 2022 Feb 14;10:826615. doi: 10.3389/fchem.2022.826615 (PMC8882737; doi:10.3389/fchem.2022.826615)
Supplement: Supplementary file 1 [file DataSheet1.PDF]

Supporting Information

Antioxidant Aryl-substituted Phthalan Derivatives Produced by Endophytic Fungus *Cytospora rhizophorae*

Hongxin Liu[1], Zhaoming Liu[1], Yanjiang Zhang[2], Yuchan Chen[1], Huan Wang[2,3], Haibo Tan[2,3*], Weimin Zhang[1*]

[1]State Key Laboratory of Applied Microbiology Southern China, Guangdong Provincial Key Laboratory of Microbial Culture Collection and Application, Guangdong Open Laboratory of Applied Microbiology, Institute of Microbiology, Guangdong Academy of Sciences, Guangzhou 510070, China

[2]National Engineering Research Center of Navel Orange, Gannan Normal University, Ganzhou, 341000, China

[3]Key Laboratory of South China Agricultural Plant Molecular Analysis and Genetic Improvement, Guangdong Provincial Key Laboratory of Applied Botany, South China Botanical Garden, Chinese Academy of Sciences, Guangzhou 510650, China

*** Correspondence:**

Haibo Tan

tanhaibo@scbg.ac.cn

Weimin Zhang

wmzhang@gdim.cn

Contents

S1. General Experimental Procedures......S4

S2. Computational methods......S4

Figure S1. ^{1}H NMR spectrum (500 MHz, CD_3COCD_3) of **1**......S8

Figure S2. ^{13}C NMR spectrum (125 MHz, CD_3COCD_3) of **1**......S8

Figure S3. ^{1}H-^{1}H COSY spectrum (500 MHz, CD_3COCD_3) of **1**......S9

Figure S4. HSQC spectrum of **1**......S9

Figure S5. HMBC spectrum of **1**......S10

Figure S6. NOESY spectrum of **1**......S10

Figure S7. HRESIMS spectrum of **1**......S11

Figure S8. UV spectrum of **1**......S11

Figure S9. CD spectrum of **1**......S12

Figure S10. IR spectrum of **1**......S12

Figure S11. ^{1}H NMR spectrum (500 MHz, CD_3COCD_3) of **2**......S13

Figure S12. ^{13}C NMR spectrum (125 MHz, CD_3COCD_3) of **2**......S13

Figure S13. ^{1}H-^{1}H COSY spectrum (500 MHz, CD_3COCD_3) of **2**......S14

Figure S14. HSQC spectrum of **2**......S14

Figure S15. HMBC spectrum of **2**......S15

Figure S16. HRESIMS spectrum of **2**......S15

Figure S17. UV spectrum of **2**......S16

Figure S18. CD spectrum of **3**......S16

Figure S19. IR spectrum of **3**......S17

Figure S20. ^{1}H NMR spectrum (500 MHz, CD_3COCD_3) of **3**......S17

Figure S21. ^{13}C NMR spectrum (125 MHz, CD_3COCD_3) of **3**......S18

Figure S22. ^{1}H-^{1}H COSY spectrum (500 MHz, CD_3COCD_3) of **3**......S18

Figure S23. HSQC spectrum of **3**......S19

Figure S24. HMBC spectrum of **3**......S19

Figure S25. NOESY spectrum of **3**......S20

Figure S26. HRESIMS spectrum of **3**......S20

Figure S27. UV spectrum of **3**......S21

Figure S28. CD spectrum of **3**......S21

Figure S29. IR spectrum of **3**......S22

Figure S30. ^{1}H NMR spectrum (500 MHz, CD_3COCD_3) of **4**......S22

Figure S31. ^{13}C NMR spectrum (125 MHz, CD_3COCD_3) of **4**......S23

Figure S32. HRESIMS spectrum of **4**......S23

Figure S33. UV spectrum of **4**......S24

Figure S34. CD spectrum of **4**......S24

Figure S35. IR spectrum of **4**......S25

Figure S36. ^{1}H NMR spectrum (600 MHz, CD_3COCD_3) of **5**......S25

Figure S37. ^{13}C NMR spectrum (150 MHz, CD_3COCD_3) of **5**......S26

Figure S38. ^{1}H-^{1}H COSY spectrum (600 MHz, CD_3COCD_3) of **5**......S26

Figure S39. HSQC spectrum of **5**......S27

Figure S40. HMBC spectrum of **5**......S27

Figure S41. HRESIMS spectrum of **5**......S28

Figure S42. UV spectrum of **5**......S28

Figure S43. CD spectrum of **5**......S29

Figure S44. IR spectrum of **5**......S29

Figure S45. ^{1}H NMR spectrum (600 MHz, CD_3COCD_3) of **6**......S30

Figure S46. ^{13}C NMR spectrum (150 MHz, CD_3COCD_3) of **6**......S30

Figure S47. ^{1}H-^{1}H COSY spectrum (600 MHz, CD_3COCD_3) of **6**......S31

Figure S48. HSQC spectrum of **6**......S31

Figure S49. HMBC spectrum of **6**......S32

Figure S50. NOESY spectrum of **6**......S32

Figure S51. HRESIMS spectrum of **6**......S33

Figure S52. UV spectrum of **6**......S33

Figure S53. CD spectrum of **6**......S34

Figure S54. IR spectrum of **6**......S34

S1. General Experimental Procedures

Optical rotation was measured on an Anton Paar MCP-500 spectropolarimeter (Anton Paar, Graz, Austria). UV spectra were measured on a SHIMADZU UV-2600 UV-visible spectrophotometer (Shimadzu, Kyoto, Japan). Circular dichroism (CD) spectra were obtained under N_2 gas on a Jasco 820 spectropolarimeter (Jasco Corporation, Kyoto, Japan). 1D and 2D NMR spectra were recorded on a Bruker Avance-500 and Bruker Avance-600 spectrometers (Bruker, Fällanden, Switzerland) with TMS as internal standard, δ in ppm, J in Hz. ESIMS data were collected on an Agilent Technologies 1290-6430A Triple Quad LC/MS (Agilent Technologies, Palo Alto, CA, USA). HRESIMS were done with a Thermo MAT95XP high resolution mass spectrometer (Thermo Fisher Scientific, Bremen, Germany). A Shimadzu LC-20AT (Shimadzu Corporation, Kyoto, Japan) equipped with an SPD-M20A PDA detector (Shimadzu Corporation, Kyoto, Japan) was used for HPLC separation. A YMC-pack ODS-A column (250 × 10 mm, 5 μm) was used for semipreparative HPLC separation, and a CHIRALPAK IC column (250 × 10 mm, 5 μm) column for chiral semipreparative HPLC separation. Silica gel (200-300 mesh) was used for column chromatography, and precoated silica gel GF_{254} plates (Qingdao Marine Chemical Inc., Qingdao, China) were used for TLC spotting. C_{18} reversed-phase silica gel (40-63 μm, Merck, German), and Sephadex LH-20 gel (Pharmacia Fine Chemical Co. Ltd., Sweden) were also used for column chromatography. TLC spots were visualized under UV light and by dipping into 10% H_2SO_4 in alcohol followed by heating.

S2. Computational methods

Methods. Merck molecular force field (MMFF) and DFT/TD-DFT calculations were carried out with the Spartan'14 software (Wavefunction Inc., Irvine, CA, USA) and the Gaussian 09 program, respectively [1]. Conformers within the 10 kcal mol^{-1} energy window were generated and optimized using DFT calculations at the b3lyp/def2svp or b3lyp/6-31+g(d,p) level. Frequency calculations were performed at the same level to confirm that each optimized conformer was true minimum and to estimate their relative thermal free energy (ΔG) at 298.15 K. Conformers with the Boltzmann distribution over 5% were chosen for ECD calculations in methanol at the cam-b3lyp/def2svp or b3lyp/6-311+g(d,p) level. Thirty excited state were chosen. Solvent effects were taken into consideration using the self-consistent reaction field (SCRF) method with the polarizable continuum model (PCM) [2]. The ECD spectrum was generated by the SpecDis program [3] using a Gaussian band shape with 0.30 eV exponential half-width from dipole-length dipolar and rotational strengths.

References

[1] M.J. Frisch, G.W. Trucks, H.B. Schlegel, G.E. Scuseria, M.A. Robb, J R. Cheeseman, G. Scalmani, V. Barone, B. Mennucci, G.A. Petersson, H. Nakatsuji, M. Caricato, X. Li, H.P. Hratchian, A.F. Izmaylov, J. Bloino, G. Zheng, J.L. Sonnenberg, M. Hada, M. Ehara, K. Toyota, R. Fukuda, J. Hasegawa, M. Ishida, T. Nakajima, Y. Honda, O.

Kitao, H. Nakai, T. Vreven, J.A. Montgomery Jr., J.E. Peralta, F. Ogliaro, M. Bearpark, J.J. Heyd, E. Brothers, K. N. Kudin, V.N. Staroverov, R. Kobayashi, J. Normand, K. Raghavachari, A. Rendell, J.C. Burant, S.S. Iyengar, J. Tomasi, M. Cossi, N. Rega, J.M. Millam, M. Klene, J.E. Knox, J.B. Cross, V. Bakken, C. Adamo, J. Jaramillo, R. Gomperts, R.E. Stratmann, O. Yazyev, A.J. Austin, R. Cammi, C. Pomelli, J.W. Ochterski, R.L. Martin, K. Morokuma, V.G. Zakrzewski, G.A. Voth, P. Salvador, J.J. Dannenberg, S. Dapprich, A.D. Daniels, Ö. Farkas, J.B. Foresman, J.V. Ortiz, J. Cioslowski, D.J. Fox, Gaussian 09, revision D.01, Gaussian, Inc., Wallingford, CT, 2013.

[2] P. Wu, J. Xue, L. Yao, L. Xu, H. Li, X. Wei, Org. Lett. 17 (2015) 4922.

[3] T. Bruhn, A. Schaumlöffel, Y. Hemberger, G. Bringmann, Chirality 25 (2013) 243.

Table S1. Energy analysis for the Conformers of **1** and **2**.

compounds	Conformation	G (Hartree)	G (Kcal/mol)	ΔG (Kcal/mol)	Boltzmann Dist (%)
**7*S*2'*R***	**7*S*2'*R* -a**	-1299.78967217	-815621.7887	0	71.1%
	**7*S*2'*R* -b**	-1299.78882466	-815621.2569	0.5318	28.9%

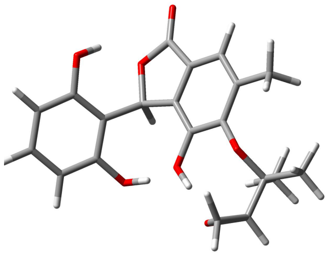

7*S*2'*R* -a

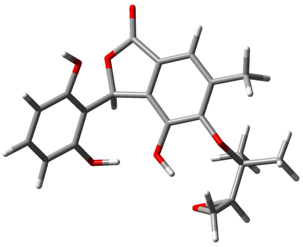

7*S*2'*R* -b

compounds	Conformation	G (Hartree)	G (Kcal/mol)	ΔG (Kcal/mol)	Boltzmann Dist (%)
**7*S*2'*S***	**7*S*2'*S* -a**	-1299.78974117	-815621.8320	0	71.1%
	**7*S*2'*S* -b**	-1299.78889211	-815621.2992	0.5329	28.9%

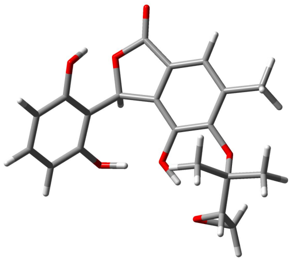

7*S*2'*S* -a

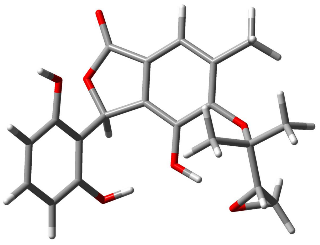

7*S*2'*S*-b

Table S2. Energy analysis for the Conformers of **3** and **4**.

compounds	Conformation	G (Hartree)	G (Kcal/mol)	ΔG (Kcal/mol)	Boltzmann Dist (%)
**7*S*2'*R***	**7*S*2'*R* -a**	-1226.76439	-769798.2125	0	100%

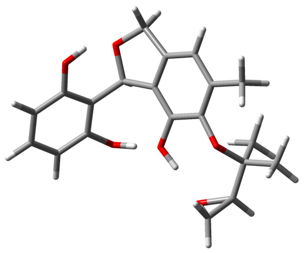

7*S*2'*R* -a

compounds	Conformation	G (Hartree)	G (Kcal/mol)	ΔG (Kcal/mol)	Boltzmann Dist (%)
**7*S*2'*S***	**7*S*2'*R* -a**	-1226.76466952	-769798.3877	0	100%

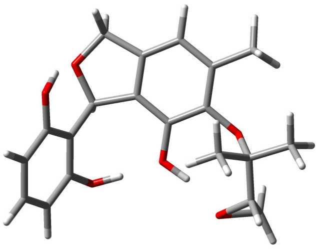

7*S*2'*S* -a

Table S3. Energy analysis for the Conformers of **5** and **6**.

compounds	Conformation	G (Hartree)	G (Kcal/mol)	ΔG (Kcal/mol)	Boltzmann Dist (%)
**7*S*2'*S***	**7*S*2'*S* -a**	-1300.836651	-816278.771	0	61.5%
**7*S*2'*S***	**7*S*2'*S* -b**	-1300.83621051	-816278.4945	0.2765	38.5%

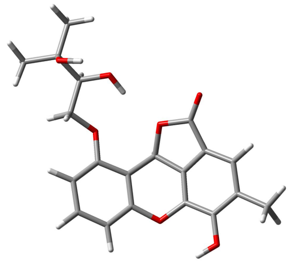

7*S*2'*S* -a

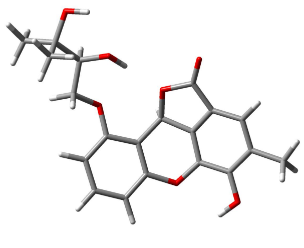

7*S*2'*S*-b

compounds	Conformation	G (Hartree)	G (Kcal/mol)	ΔG (Kcal/mol)	Boltzmann Dist (%)
**7*S*2'*R***	**7*S*2'*R* -a**	-1300.83440508	-816277.3616	0	50%
**7*S*2'*R***	**7*S*2'*R* -b**	-1300.83440508	-816277.3616	0	50%

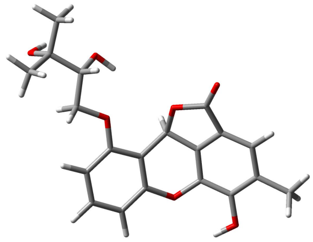

7*S*2'*R* -a

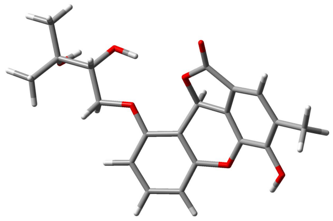

7*S*2'*R*-b

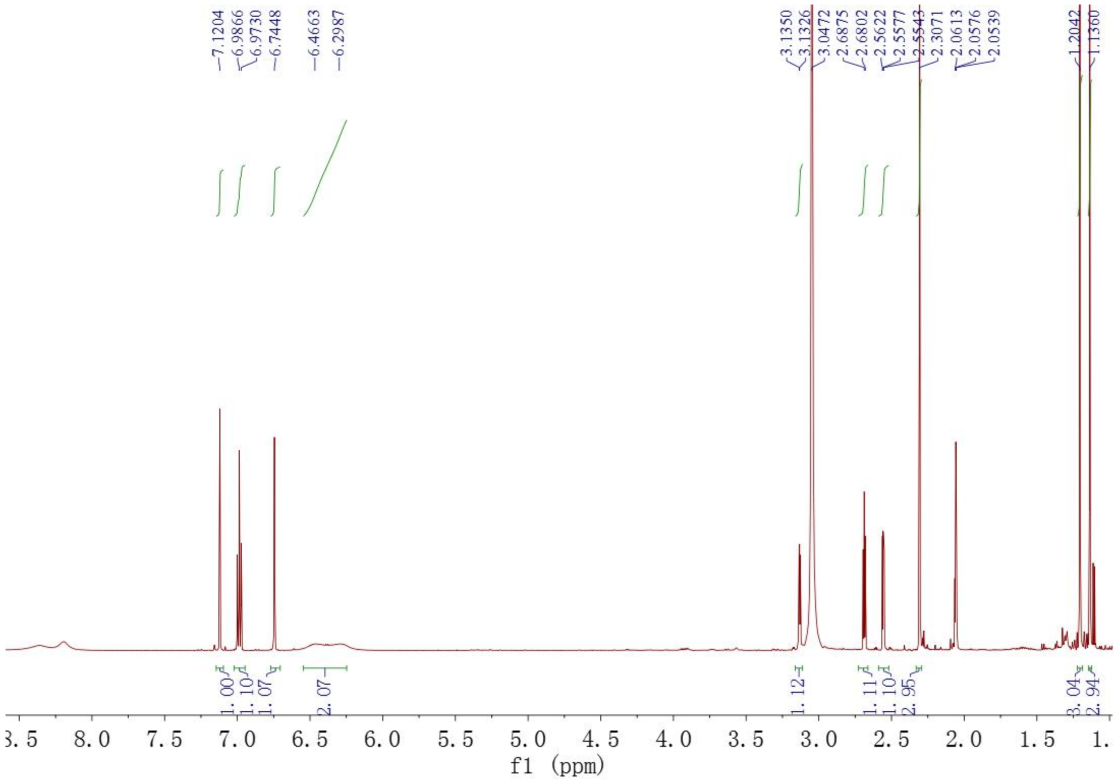

Figure S1. ^{1}H NMR spectrum (500 MHz, CD_3COCD_3) of **1**.

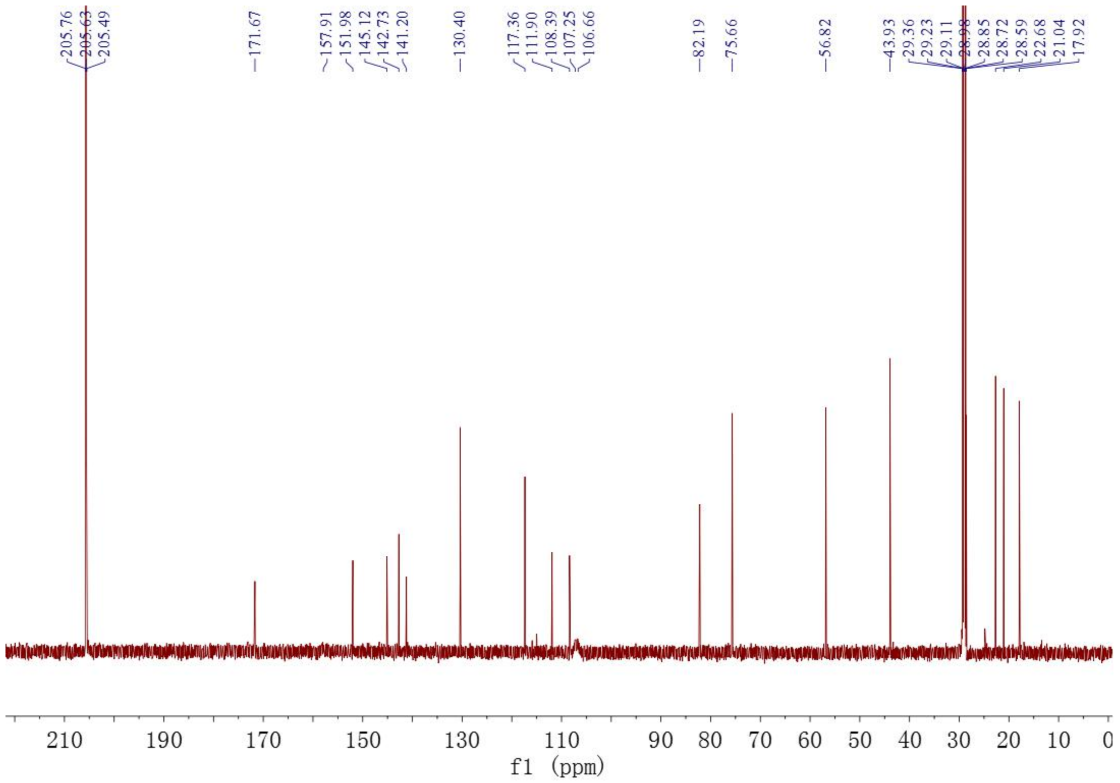

Figure S2. ^{13}C NMR spectrum (125 MHz, CD_3COCD_3) of **1**.

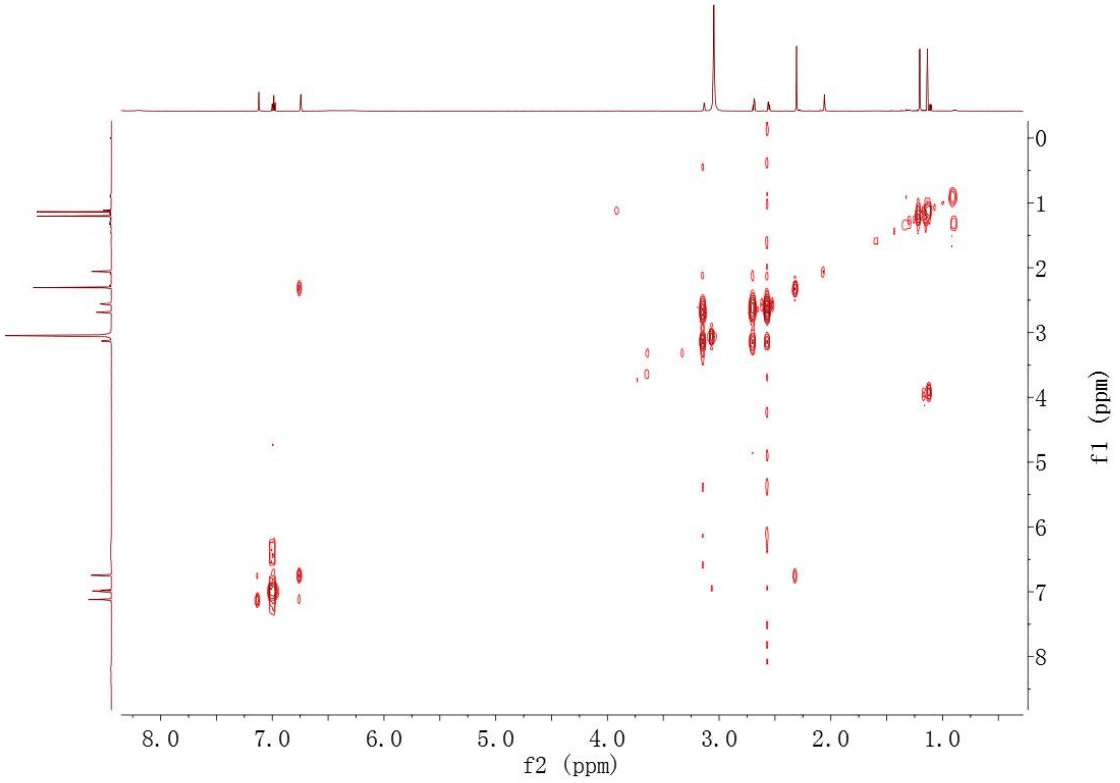

Figure S3. ^{1}H-^{1}H COSY spectrum (500 MHz, CD_3COCD_3) of **1**.

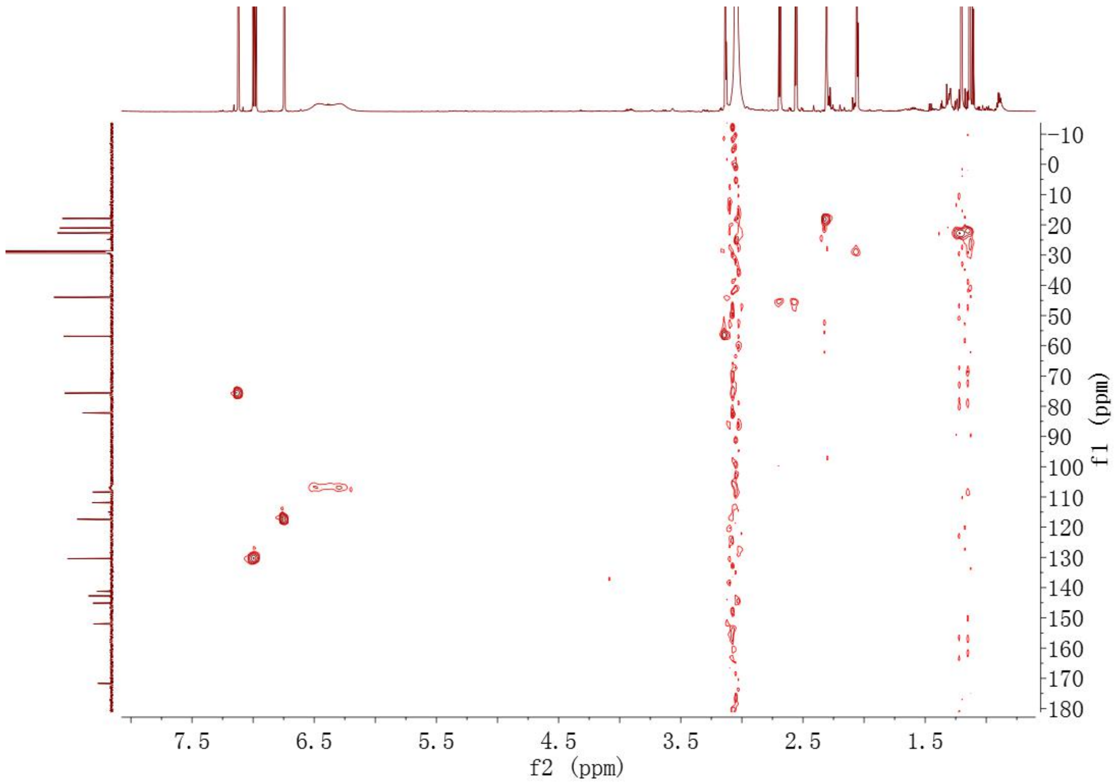

Figure S4. HSQC spectrum of **1**.

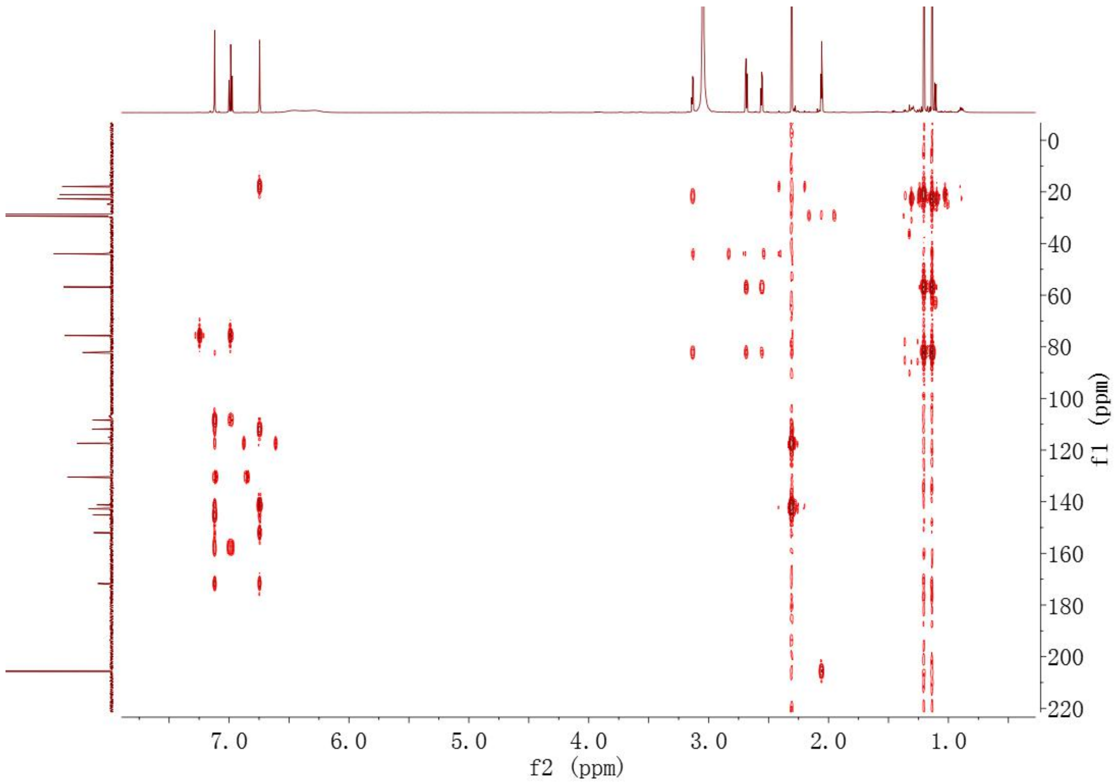

Figure S5. HMBC spectrum of **1**.

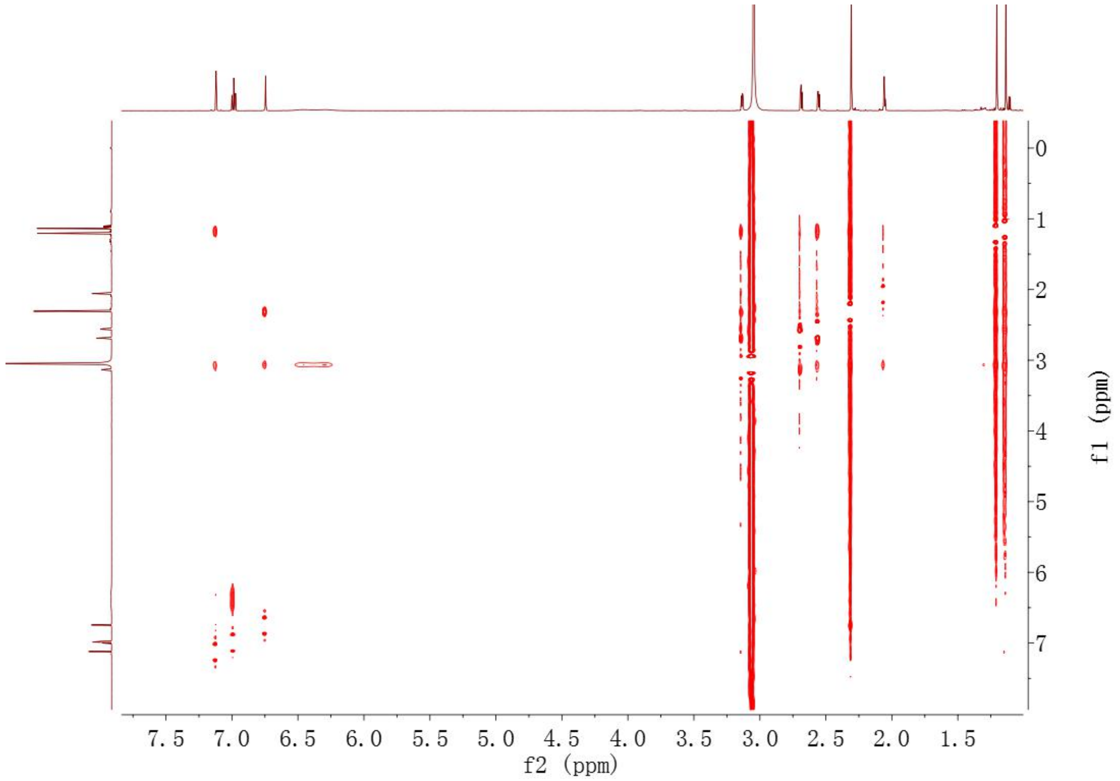

Figure S6. NOESY spectrum of **1**.

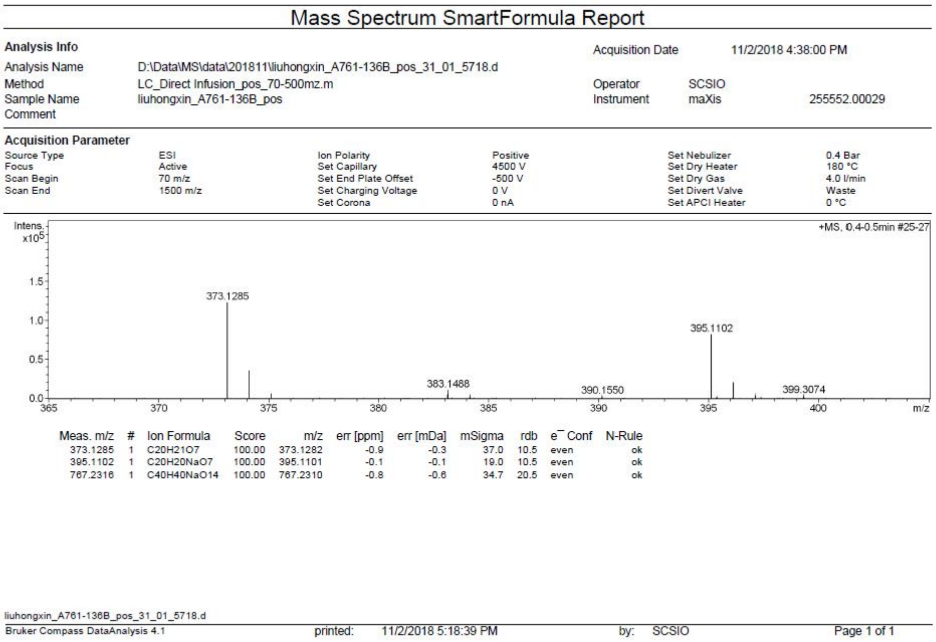

Figure S7. HRESIMS spectrum of **1**.

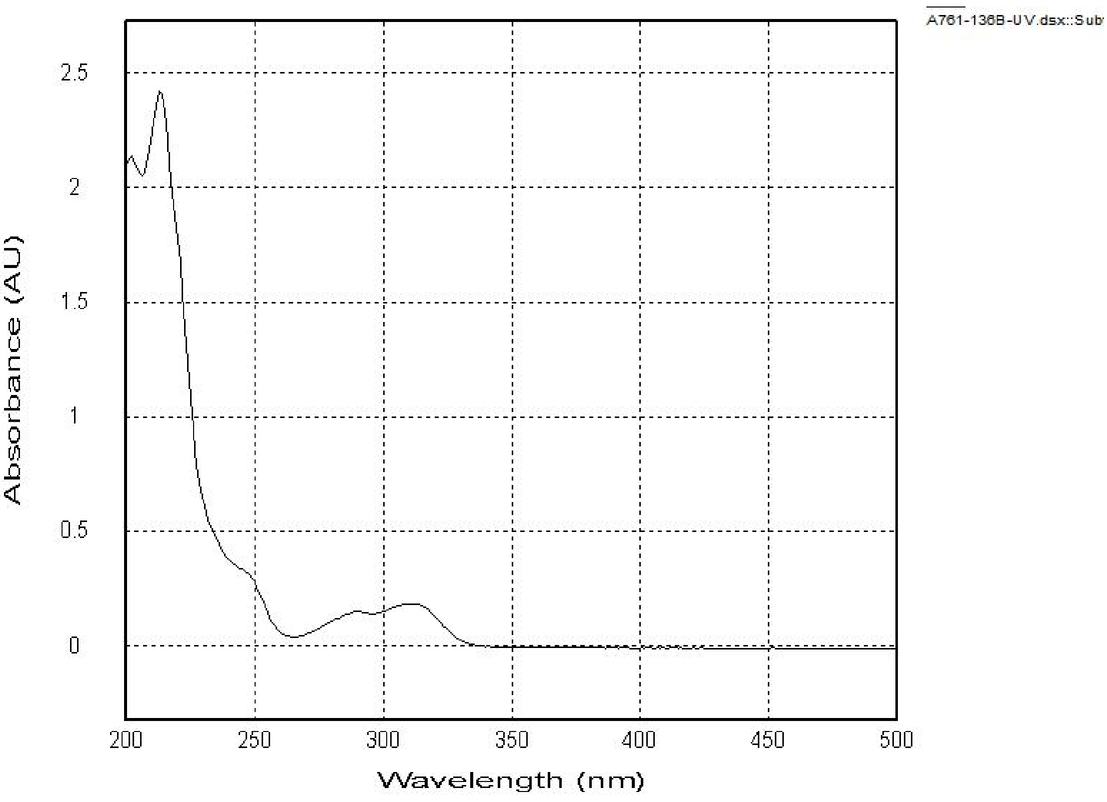

Figure S8. UV spectrum of **1**.

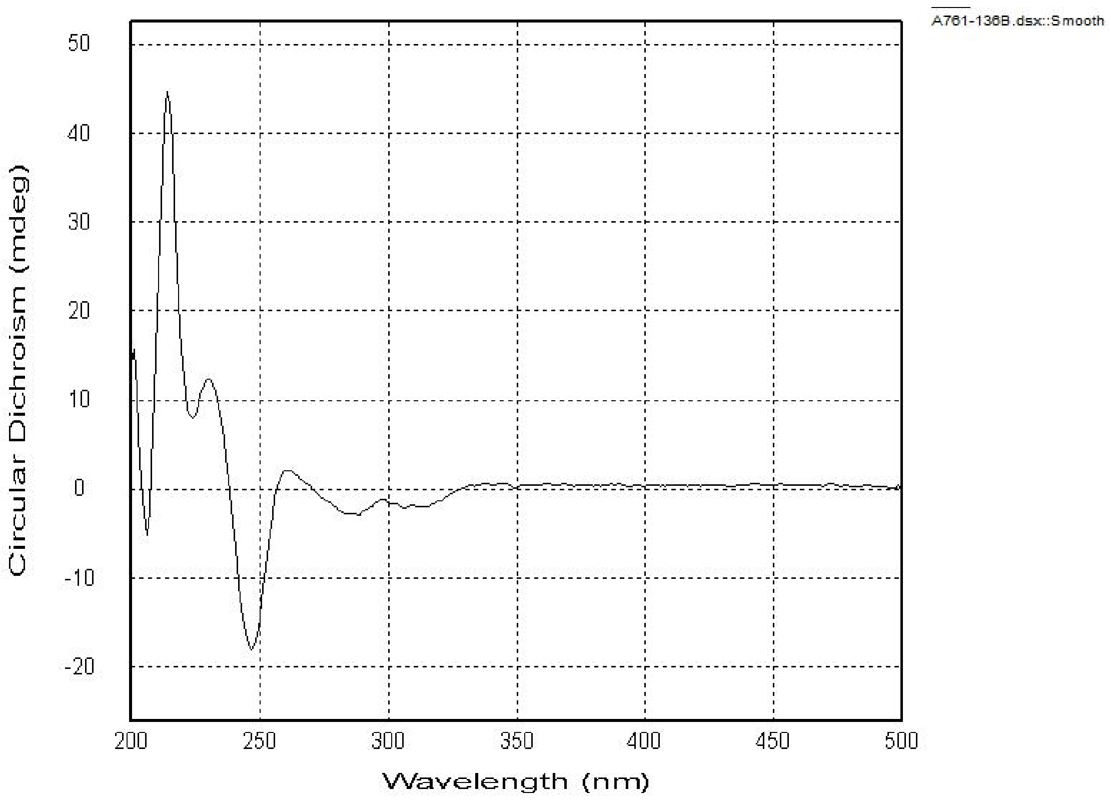

Figure S9. CD spectrum of **1**.

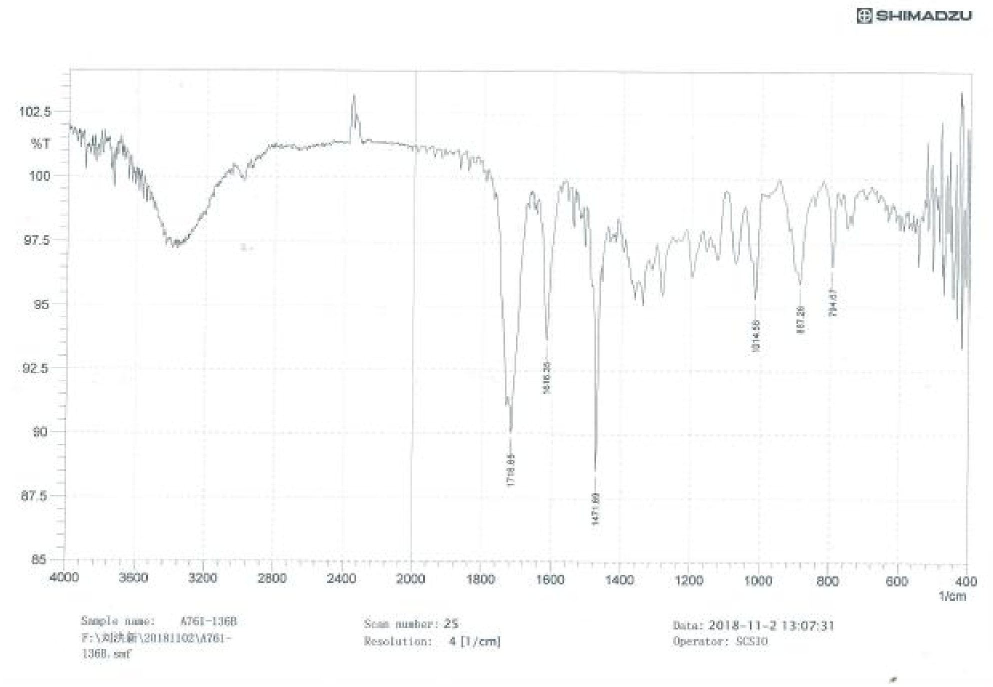

Figure S10. IR spectrum of **1**.

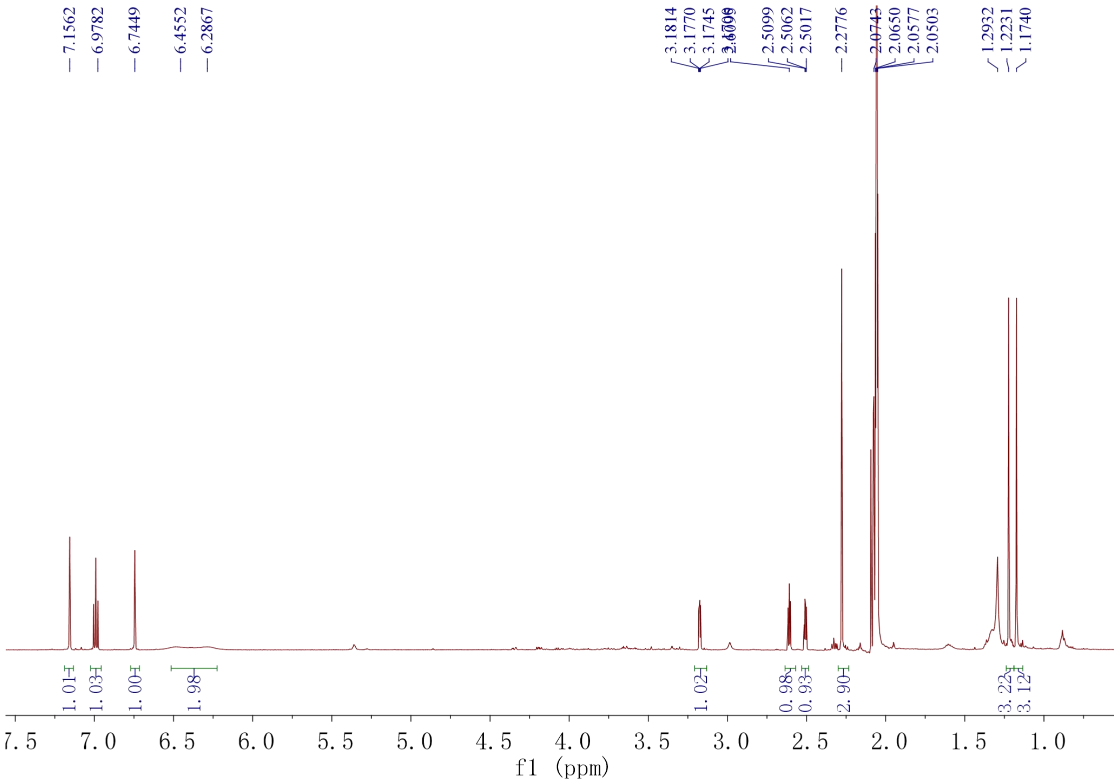

Figure S11. ^{1}H NMR spectrum (500 MHz, CD_3COCD_3) of **2**.

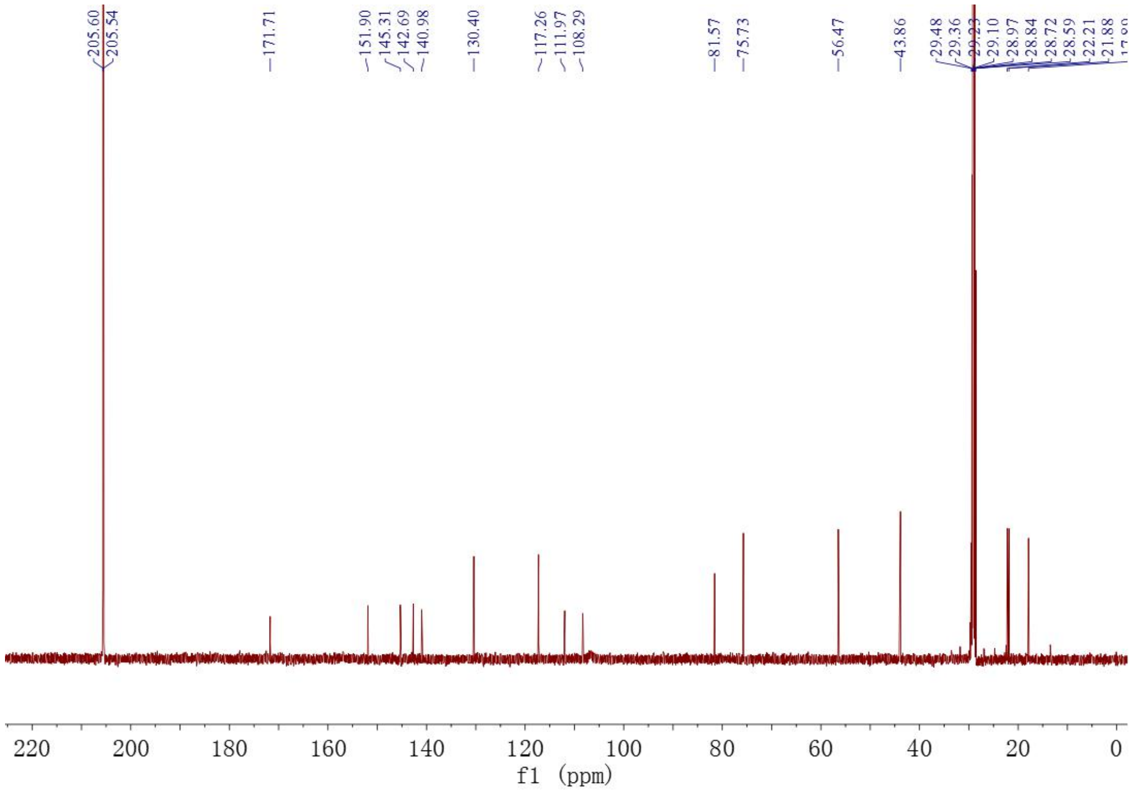

Figure S12. ^{13}C NMR spectrum (125 MHz, CD_3COCD_3) of **2**.

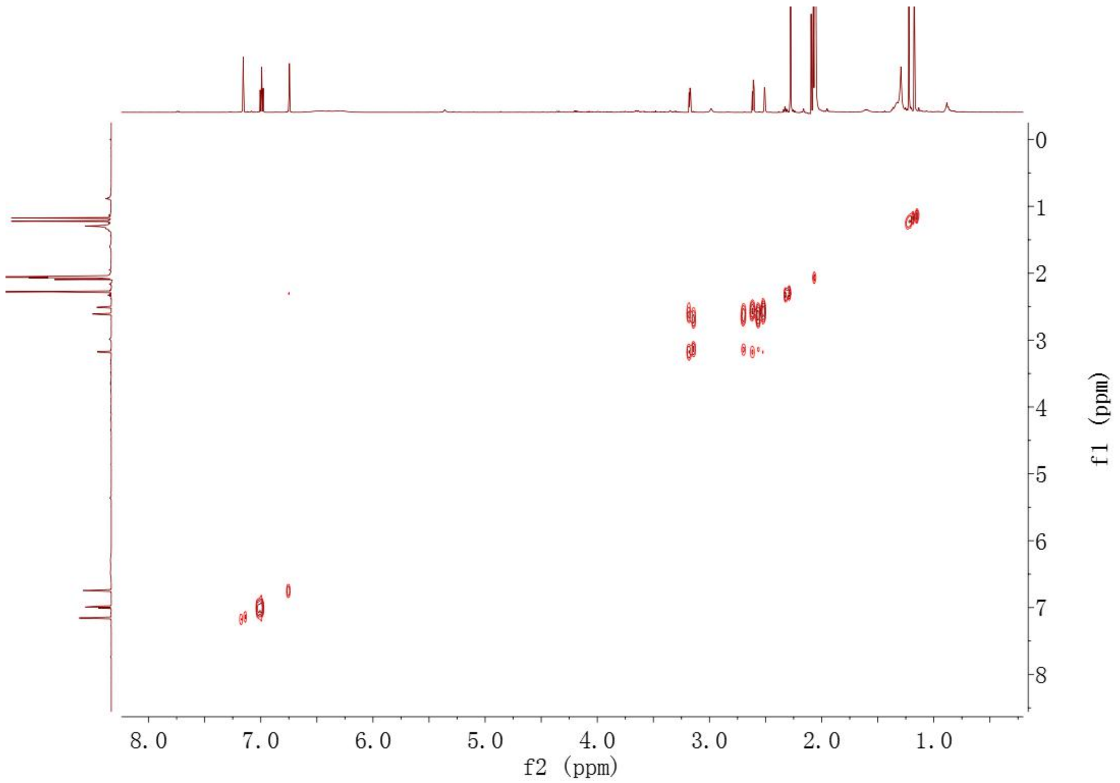

Figure S13. ^{1}H-^{1}H COSY spectrum (500 MHz, CD_3COCD_3) of **2.**

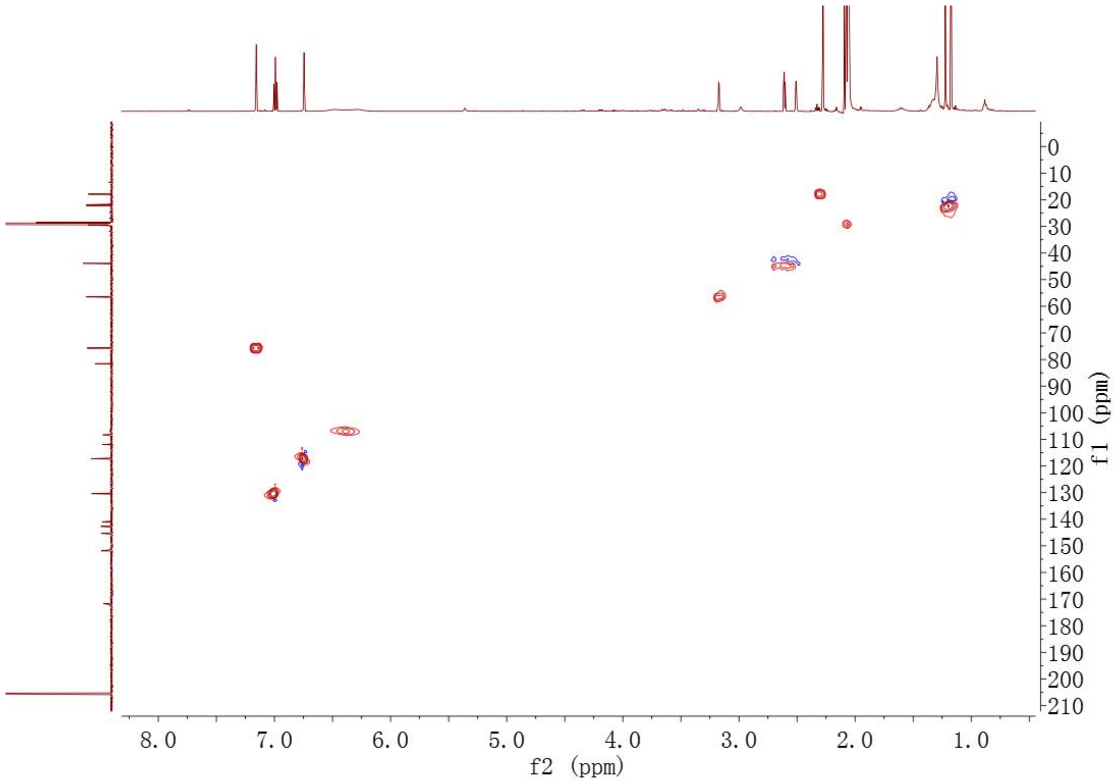

Figure S14. HSQC spectrum of **2**.

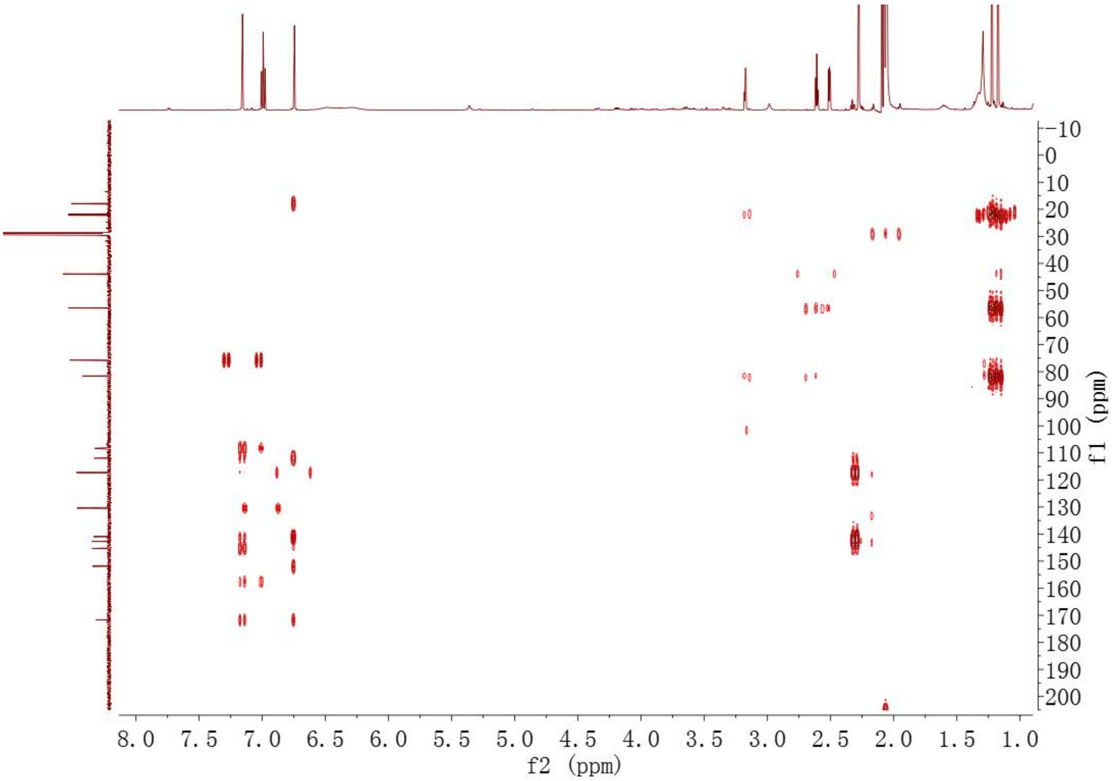

Figure S15. HMBC spectrum of **2**.

Mass Spectrum SmartFormula Report

**Analysis Info**		Acquisition Date	12/13/2018 11:54:35 AM	
Analysis Name	D:\Data\MS\data\201812\liuhongxin_A761-136A_pos_43_01_5936.d			
Method	LC_Direct Infusion_pos_70-500mz.m	Operator	SCSIO	
Sample Name	liuhongxin_A761-136A_pos	Instrument	maXis	255552.00029
Comment				

Acquisition Parameter

Source Type	ESI	Ion Polarity	Positive	Set Nebulizer	0.4 Bar
Focus	Active	Set Capillary	4500 V	Set Dry Heater	180 °C
Scan Begin	70 m/z	Set End Plate Offset	-500 V	Set Dry Gas	4.0 l/min
Scan End	1500 m/z	Set Charging Voltage	0 V	Set Divert Valve	Waste
		Set Corona	0 nA	Set APCI Heater	0 °C

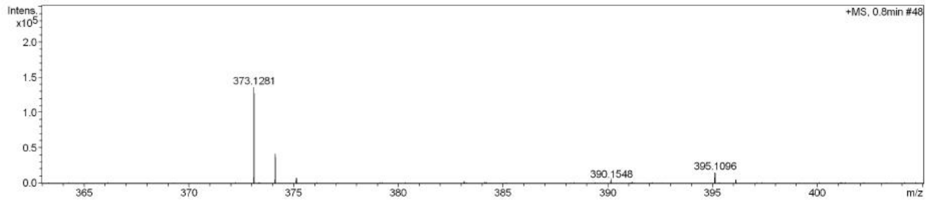

Meas. m/z	#	Ion Formula	Score	m/z	err [ppm]	err [mDa]	mSigma	rdb	e⁻ Conf	N-Rule
373.1281	1	C20H21O7	100.00	373.1282	-0.2	-0.1	44.4	10.5	even	ok
395.1096	1	C20H20NaO7	100.00	395.1101	-1.3	-0.5	48.7	10.5	even	ok
745.2487	1	C40H41O14	100.00	745.2491	-0.5	-0.4	24.4	20.5	even	ok
767.2317	1	C40H40NaO14	100.00	767.2310	0.8	0.6	18.9	20.5	even	ok

liuhongxin_A761-136A_pos_43_01_5936.d
Bruker Compass DataAnalysis 4.1 printed: 12/13/2018 2:35:34 PM by: SCSIO Page 1 of 1

Figure S16. HRESIMS spectrum of **2**.

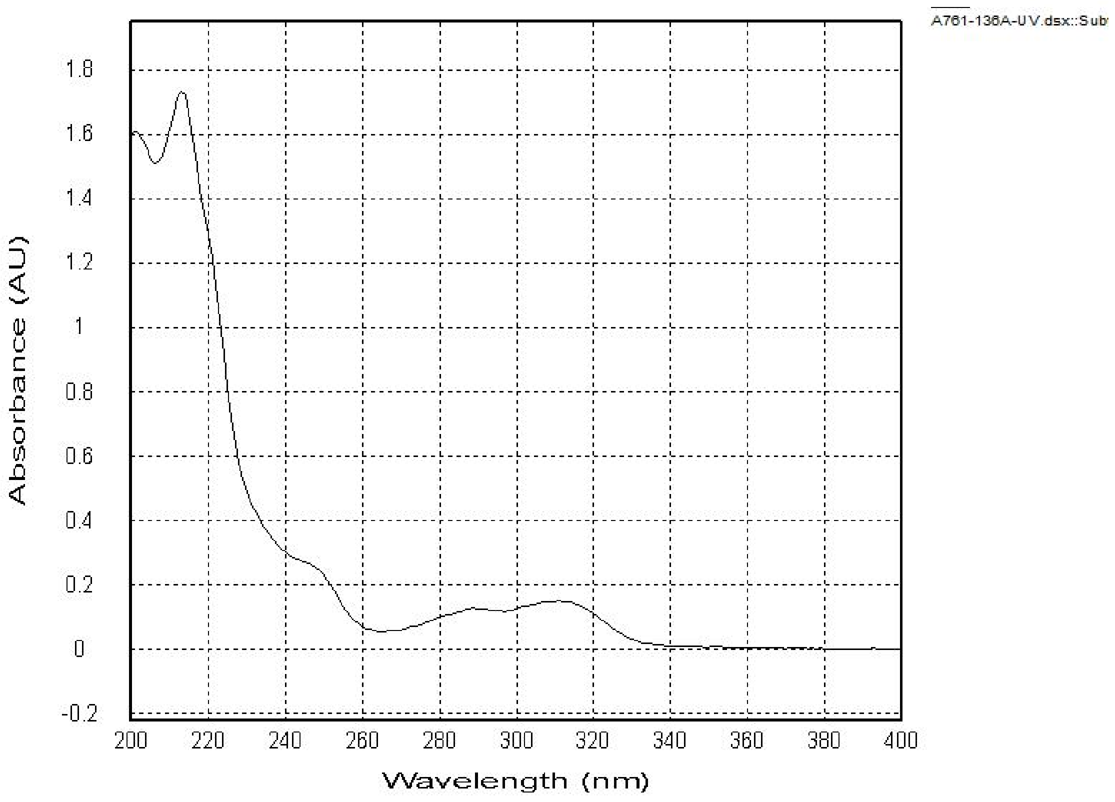

Figure S17. UV spectrum of **2**.

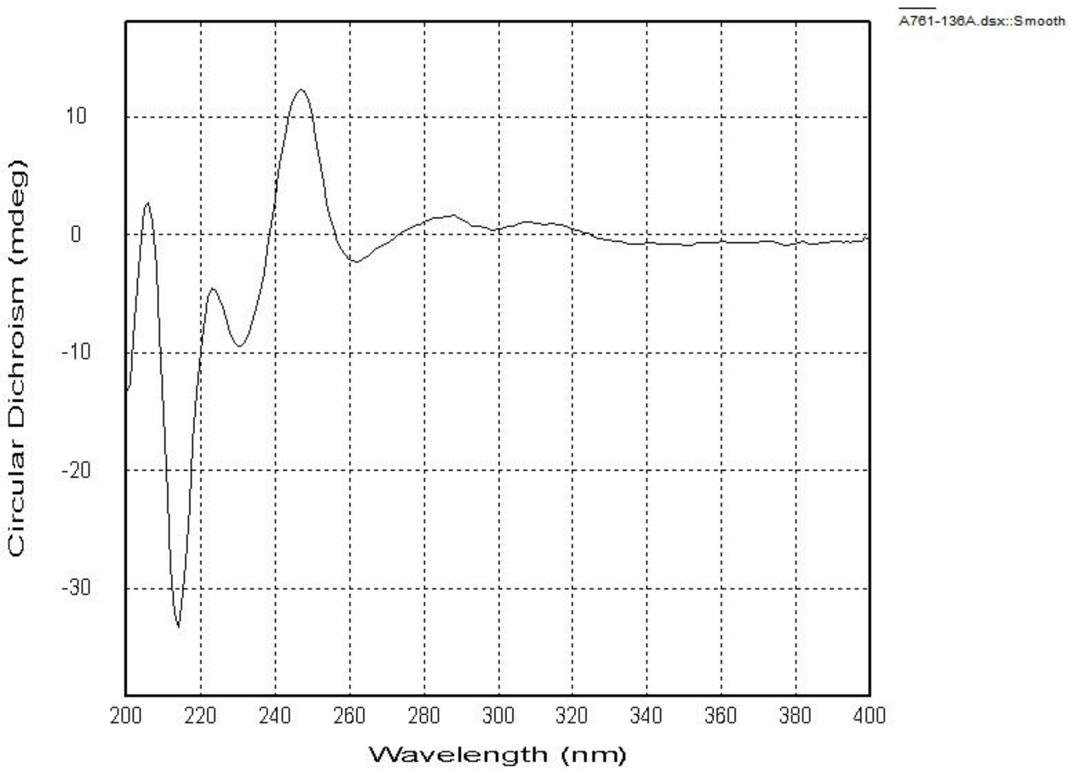

Figure S18. CD spectrum of **2**.

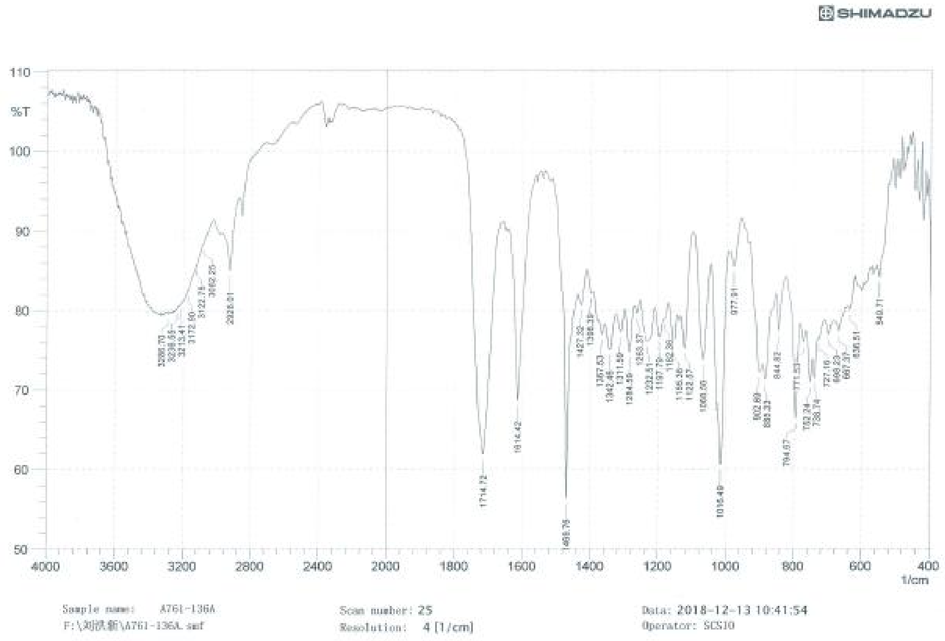

Figure S19. IR spectrum of **2**.

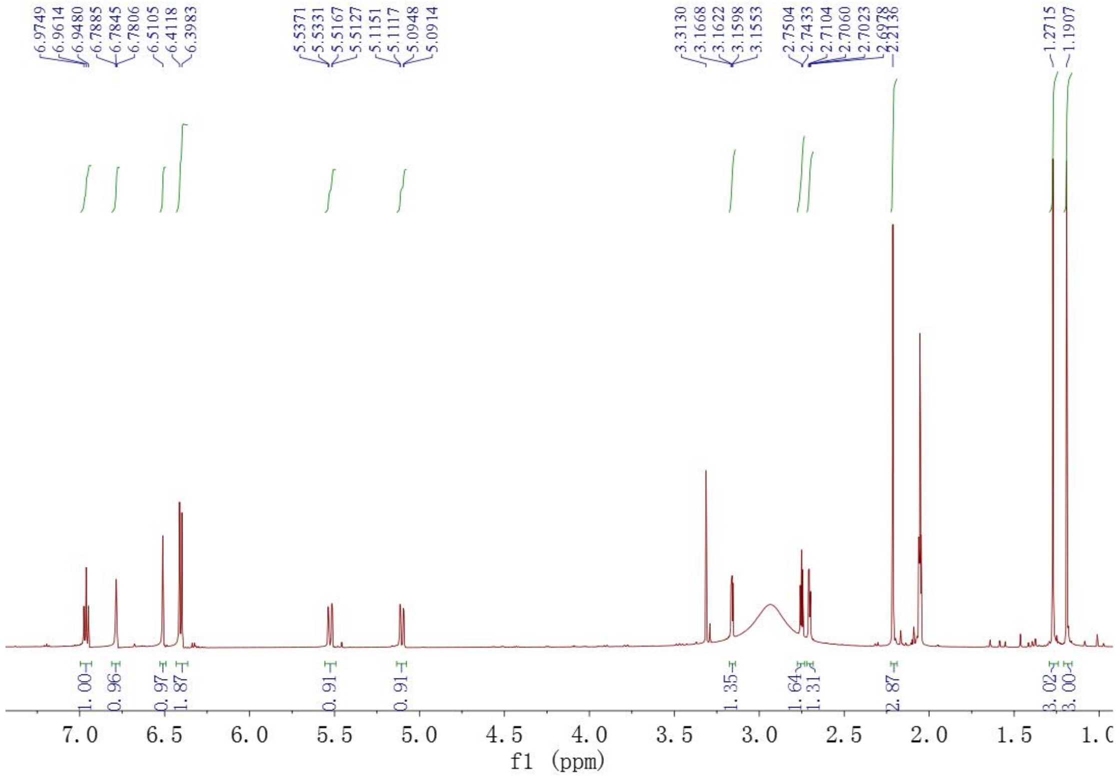

Figure S20. ^{1}H NMR spectrum (500 MHz, CD_3COCD_3) of **3**.

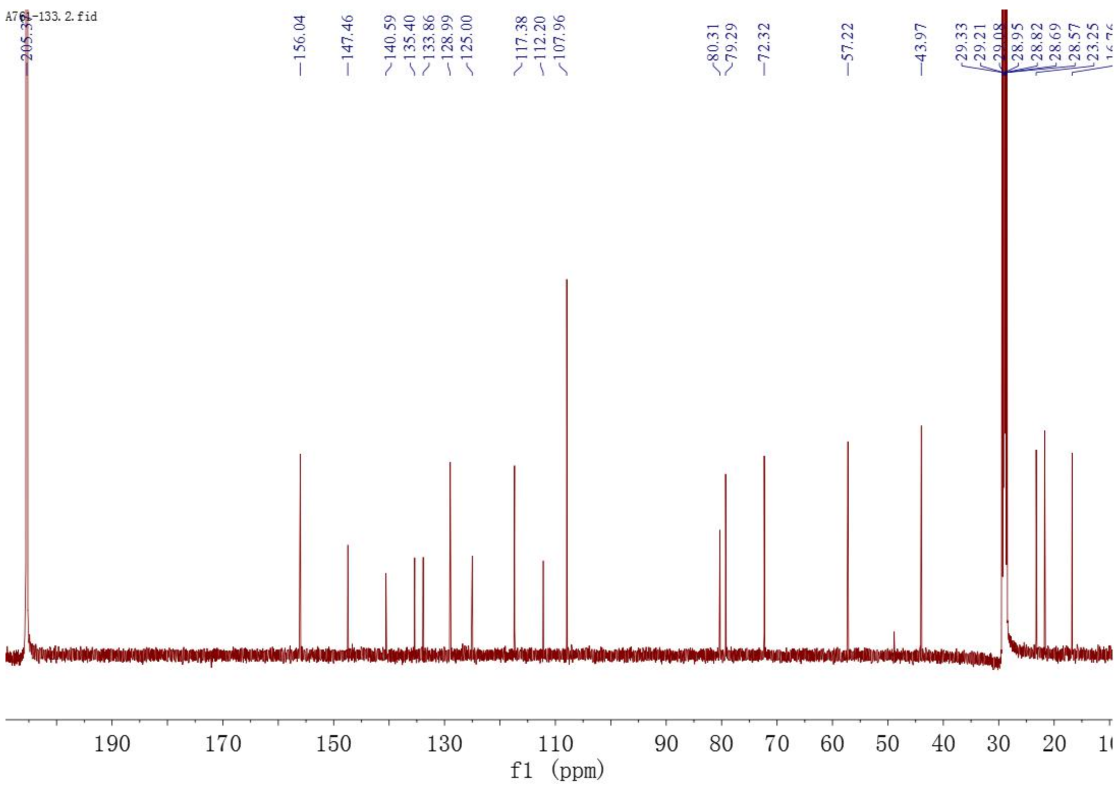

Figure S21. ^{13}C NMR spectrum (125 MHz, CD_3COCD_3) of **3**.

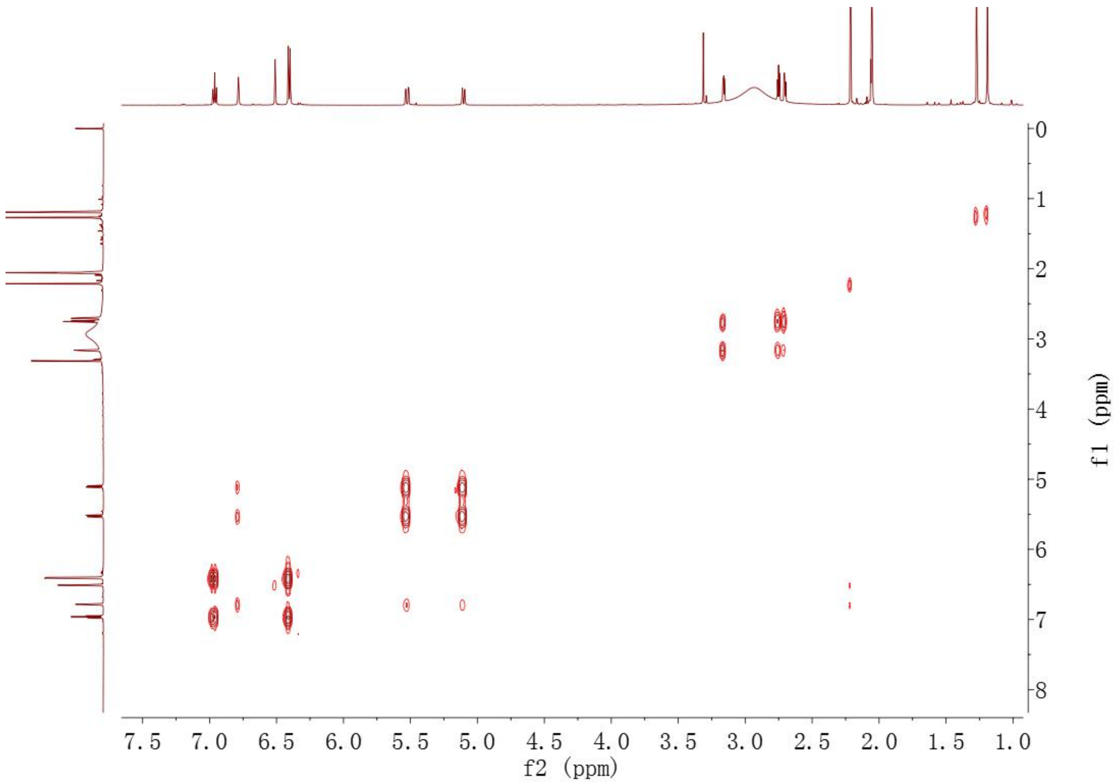

Figure S22. ^1H-^1H COSY spectrum (500 MHz, CD_3COCD_3) of **3**.

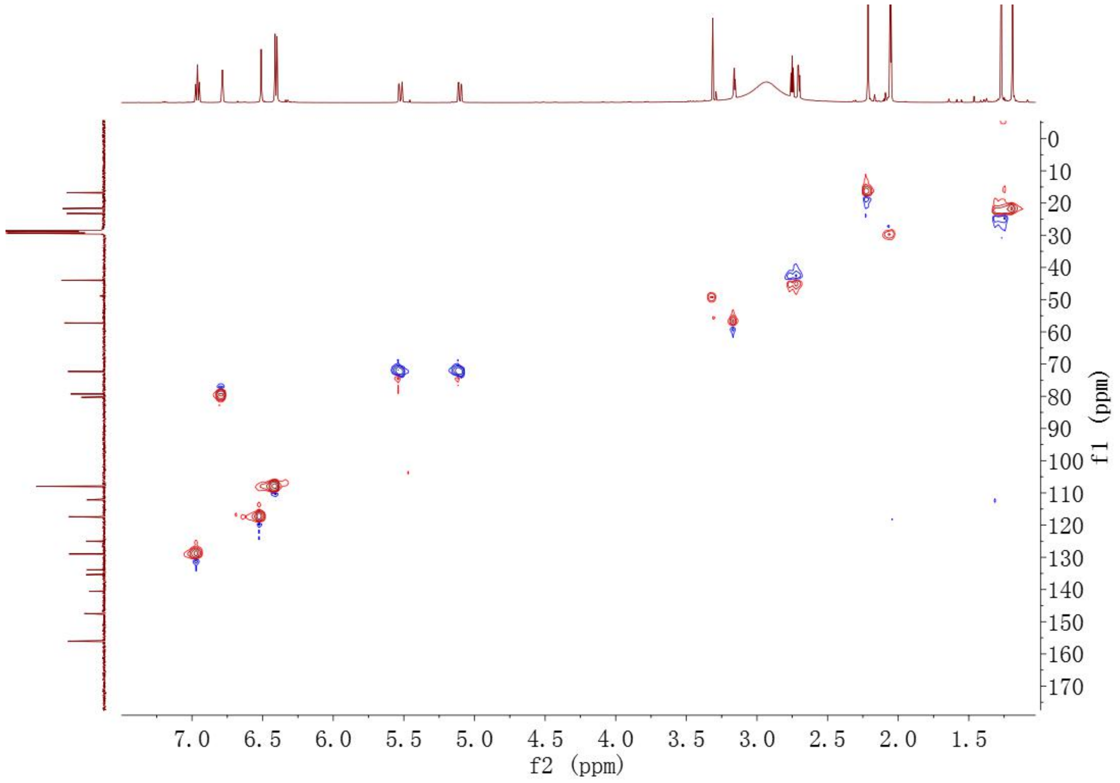

Figure S23. HSQC spectrum of **3**.

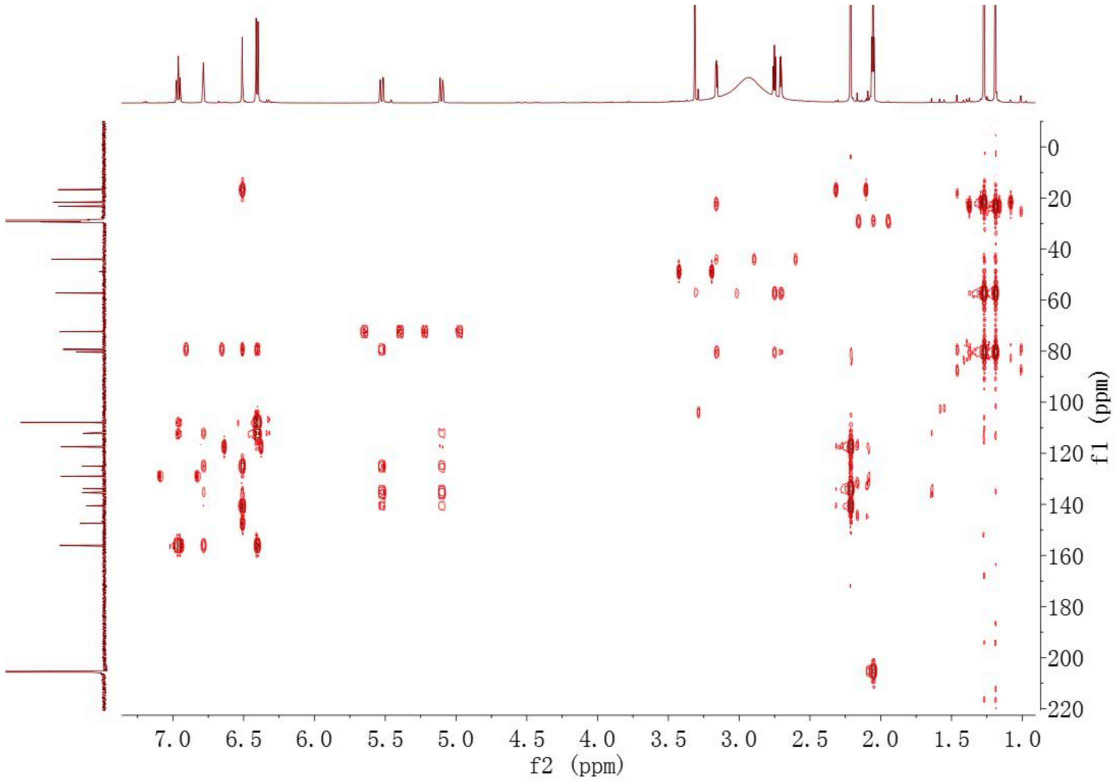

Figure S24. HMBC spectrum of **3**.

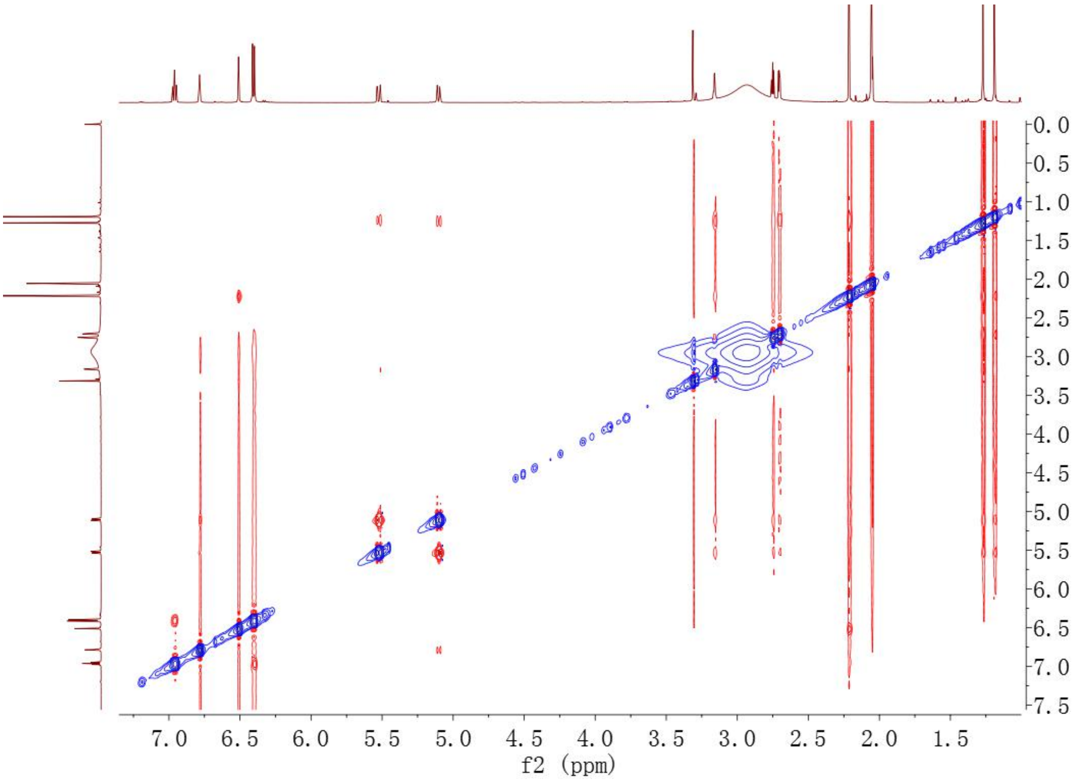

Figure S25. NOESY spectrum of **3**.

Mass Spectrum SmartFormula Report

Analysis Info

Analysis Name	D:\Data\MS\data\201811\liuhongxin_A761-133_pos_35_01_5722.d	Acquisition Date	11/2/2018 4:51:56 PM	
Method	LC_Direct Infusion_pos_70-500mz.m	Operator	SCSIO	
Sample Name	liuhongxin_A761-133_pos	Instrument	maXis	255552.00029
Comment				

Acquisition Parameter

Source Type	ESI	Ion Polarity	Positive	Set Nebulizer	0.4 Bar
Focus	Active	Set Capillary	4500 V	Set Dry Heater	180 °C
Scan Begin	70 m/z	Set End Plate Offset	-500 V	Set Dry Gas	4.0 l/min
Scan End	1500 m/z	Set Charging Voltage	0 V	Set Divert Valve	Waste
		Set Corona	0 nA	Set APCI Heater	0 °C

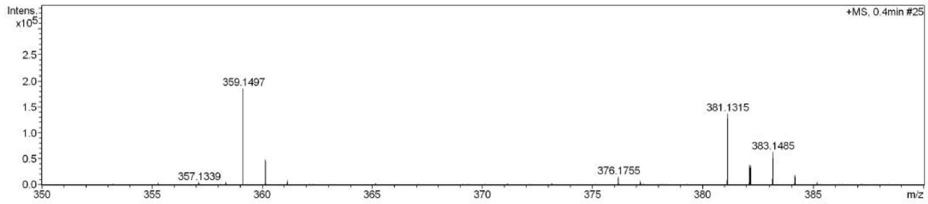

Meas. m/z	#	Ion Formula	Score	m/z	err [ppm]	err [mDa]	mSigma	rdb	e⁻ Conf	N-Rule
359.1497	1	C20H23O6	100.00	359.1489	2.1	0.8	18.9	9.5	even	ok
381.1315	1	C20H22NaO6	100.00	381.1309	1.8	0.7	33.2	9.5	even	ok
739.2727	1	C40H44NaO12	100.00	739.2725	0.2	0.2	37.5	18.5	even	ok

liuhongxin_A761-133_pos_35_01_5722.d
Bruker Compass DataAnalysis 4.1 printed: 11/2/2018 5:25:43 PM by: SCSIO Page 1 of 1

Figure S26. HRESIMS spectrum of **3**.

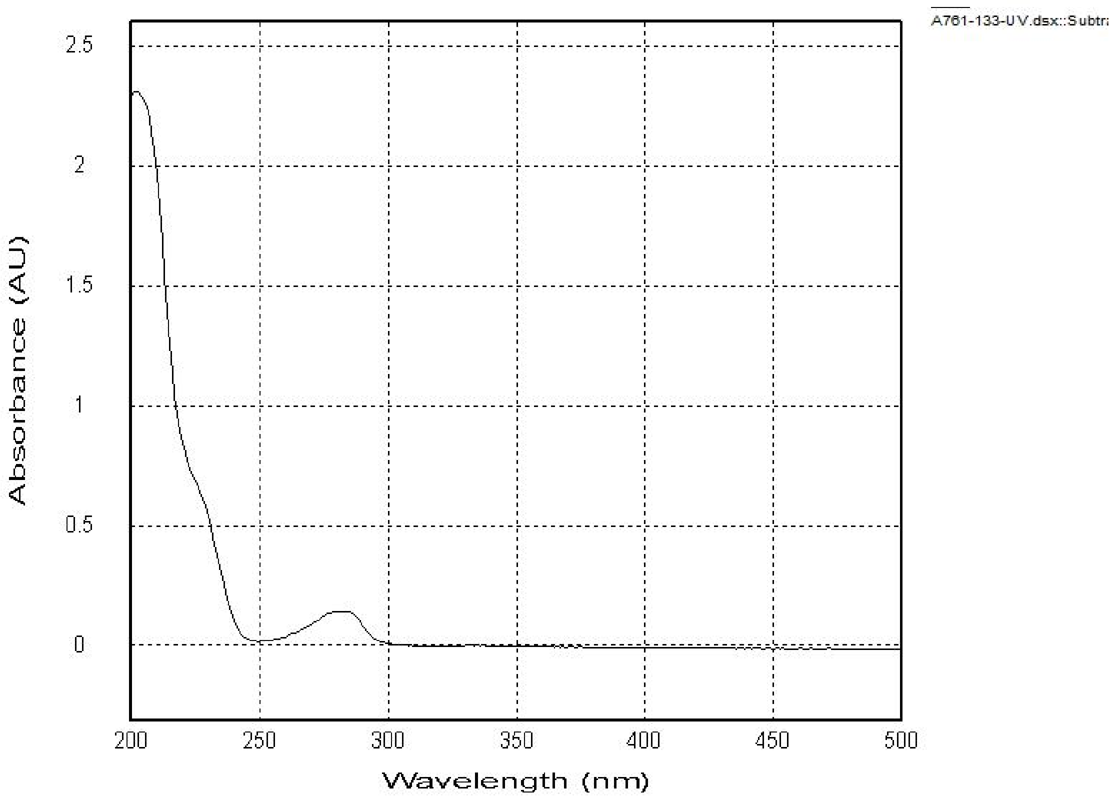

Figure S27. UV spectrum of **3**.

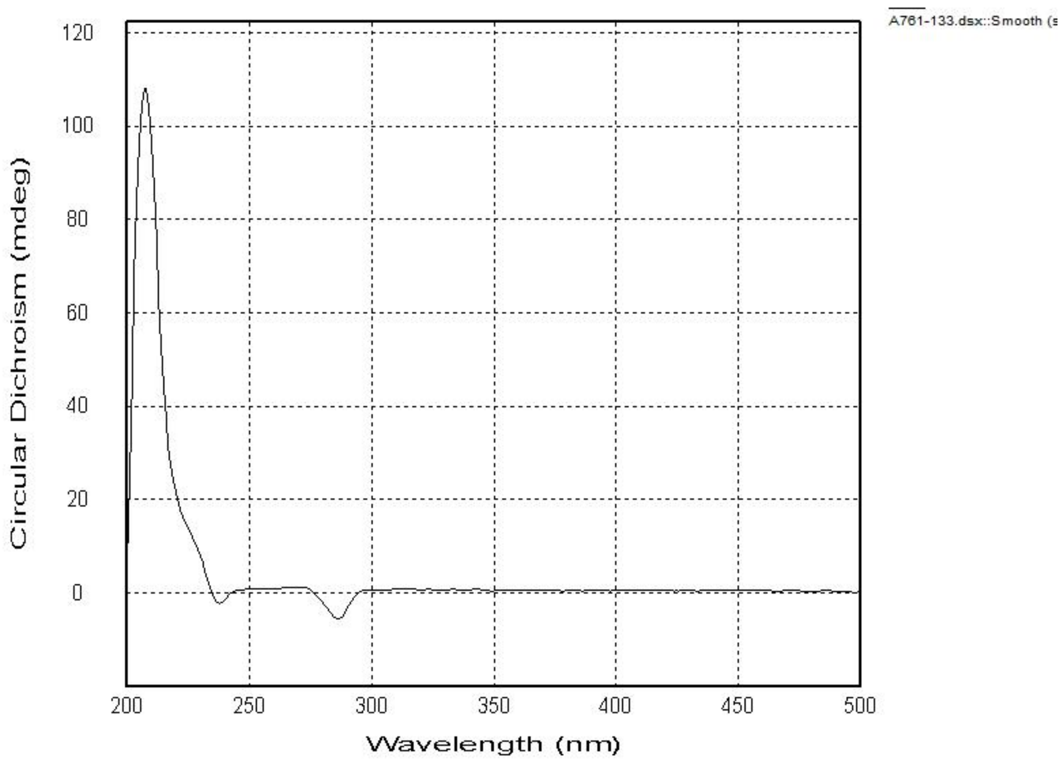

Figure S28. CD spectrum of **3**.

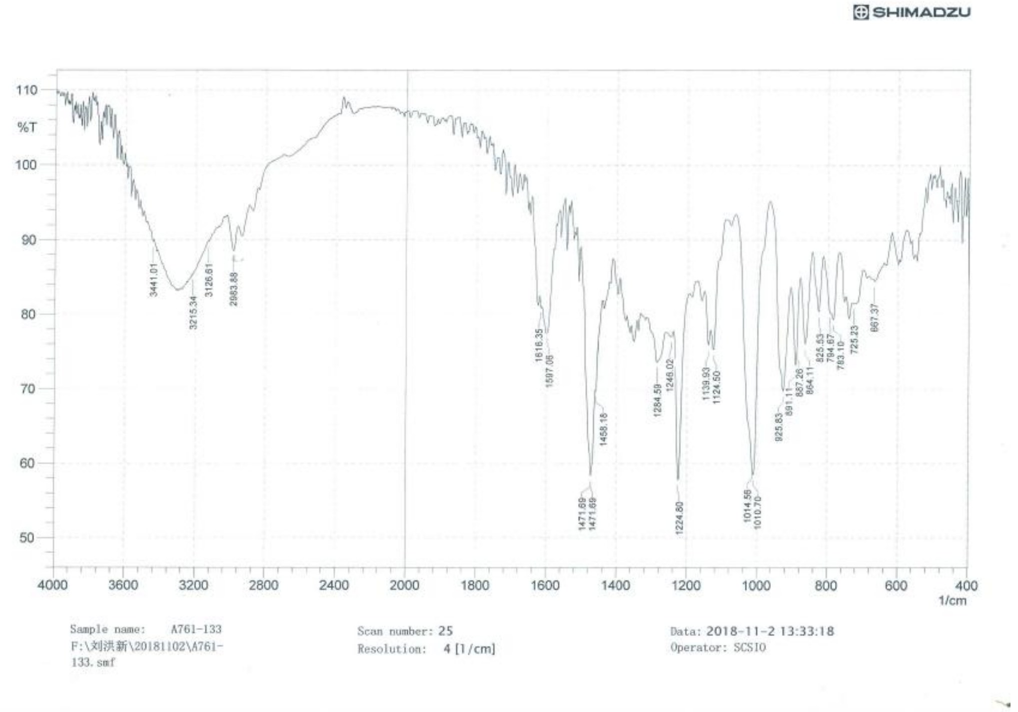

Figure S29. IR spectrum of **3**.

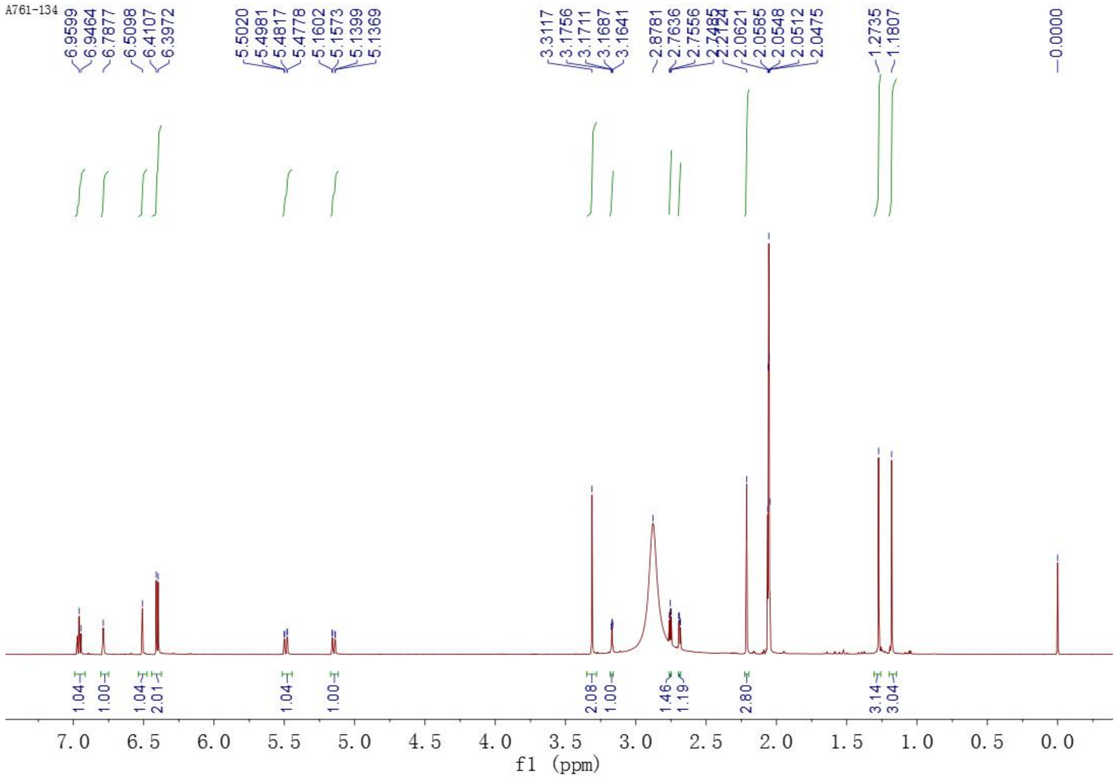

Figure S30. ^{1}H NMR spectrum (500 MHz, CD_3COCD_3) of **4**.

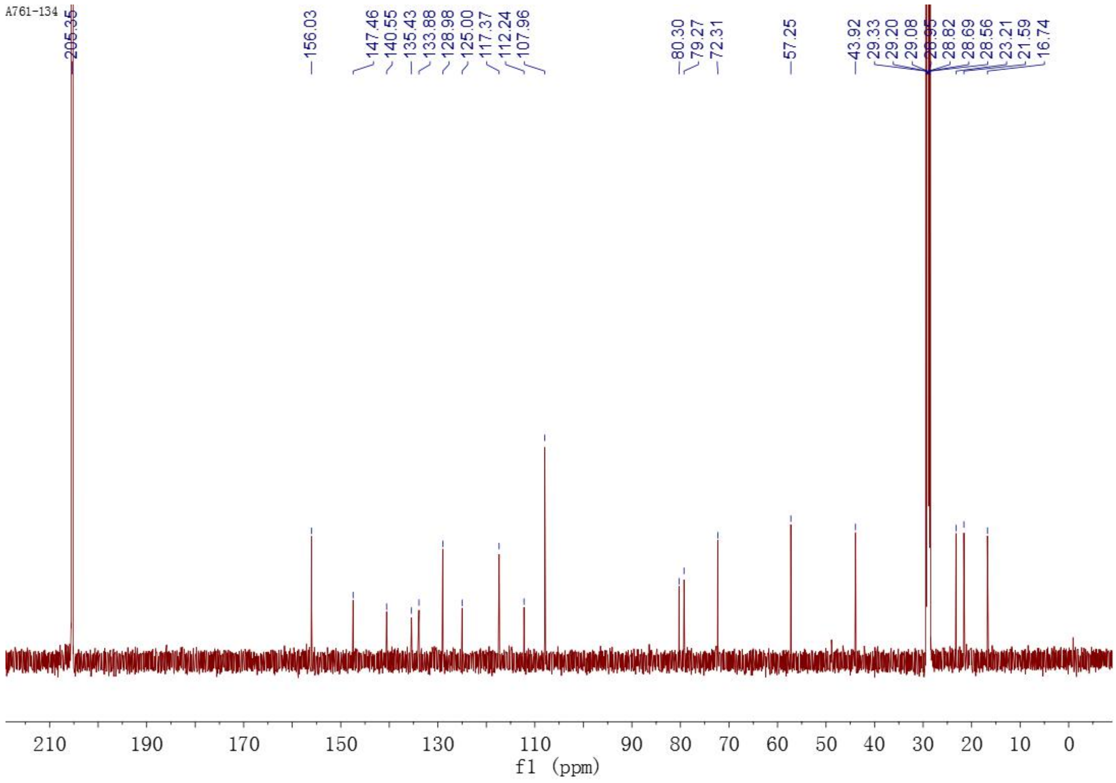

Figure S31. ^{13}C NMR spectrum (125 MHz, CD_3COCD_3) of **4**.

Mass Spectrum SmartFormula Report

Analysis Info

Analysis Name: D:\Data\MS\data\201811\liuhongxin_A761-134_pos_32_01_5719.d
Method: LC_Direct Infusion_pos_70-500mz.m
Sample Name: liuhongxin_A761-134_pos
Comment:

Acquisition Date: 11/2/2018 4:41:27 PM
Operator: SCSIO
Instrument: maXis 255552.00029

Acquisition Parameter

Source Type	ESI	Ion Polarity	Positive	Set Nebulizer	0.4 Bar
Focus	Active	Set Capillary	4500 V	Set Dry Heater	180 °C
Scan Begin	70 m/z	Set End Plate Offset	-500 V	Set Dry Gas	4.0 l/min
Scan End	1500 m/z	Set Charging Voltage	0 V	Set Divert Valve	Waste
		Set Corona	0 nA	Set APCI Heater	0 °C

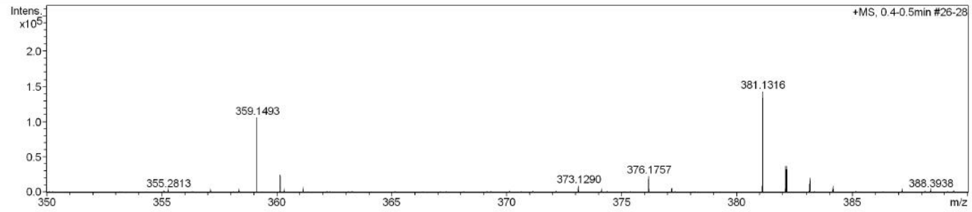

Meas. m/z	#	Ion Formula	Score	m/z	err [ppm]	err [mDa]	mSigma	rdb	e⁻ Conf	N-Rule
359.1493	1	C20H23O6	100.00	359.1489	1.1	0.4	7.4	9.5	even	ok
381.1316	1	C20H22NaO6	100.00	381.1309	1.8	0.7	55.9	9.5	even	ok
739.2725	1	C40H44NaO12	100.00	739.2725	0.0	0.0	19.0	18.5	even	ok

liuhongxin_A761-134_pos_32_01_5719.d
Bruker Compass DataAnalysis 4.1 printed: 11/2/2018 5:21:25 PM by: SCSIO Page 1 of 1

Figure S32. HRESIMS spectrum of **4**.

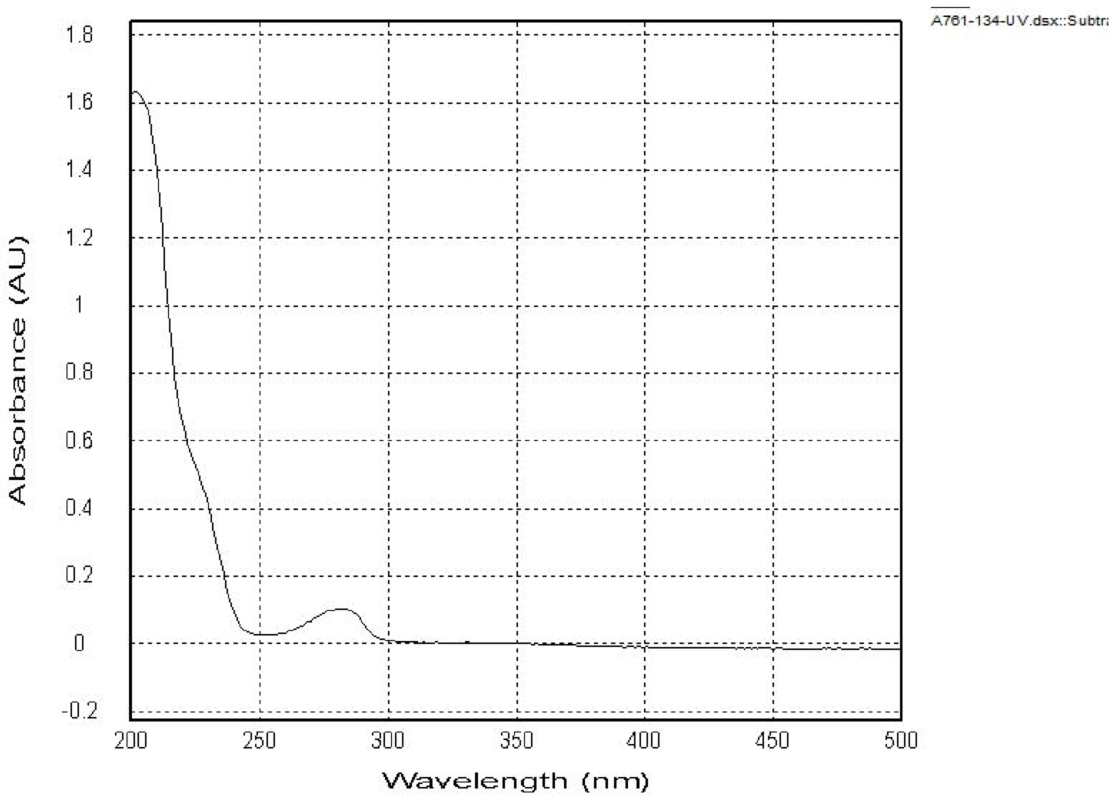

Figure S33. UV spectrum of **4**.

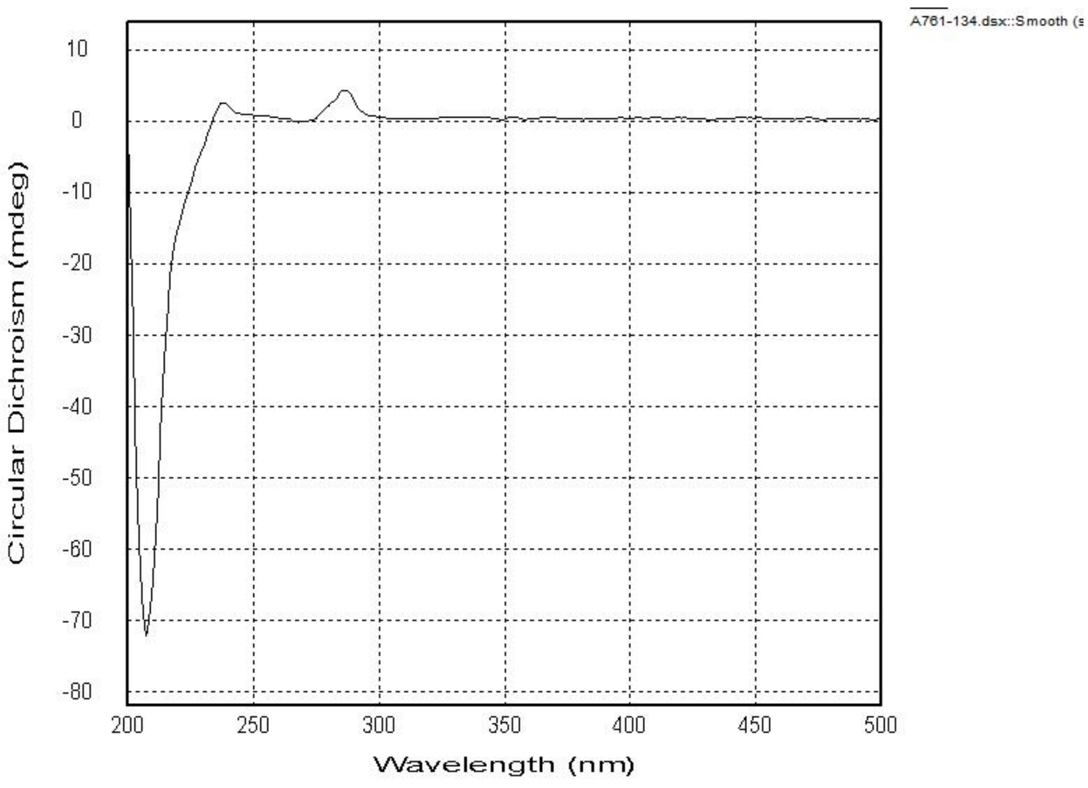

Figure S34. CD spectrum of **4**

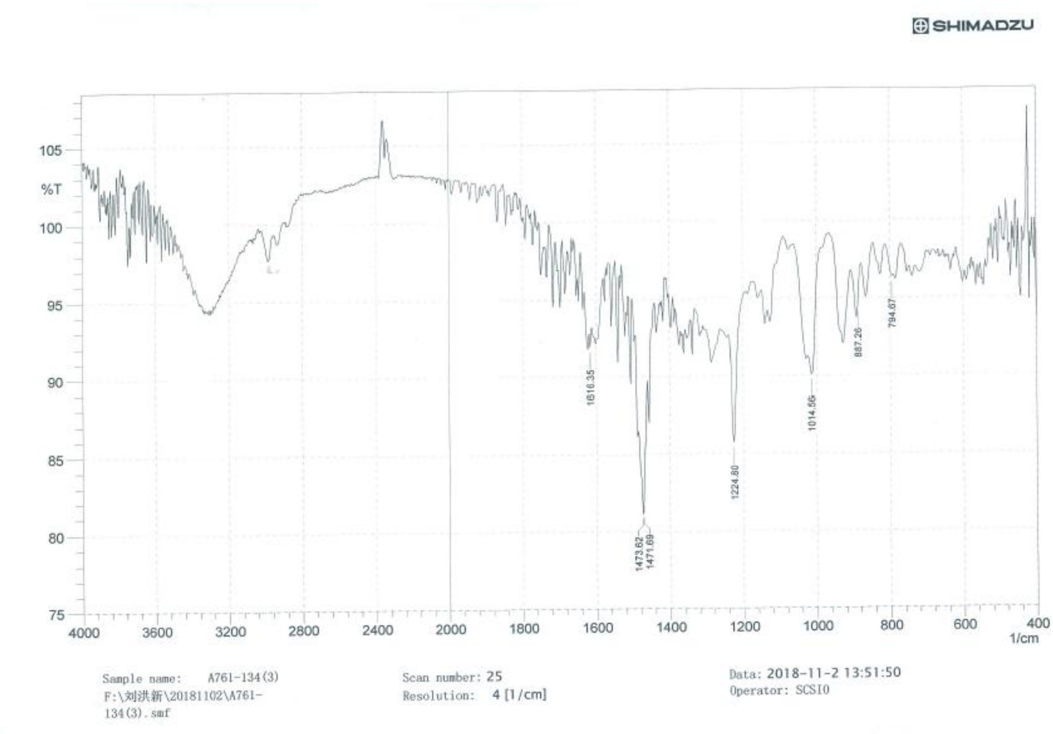

Figure S35. IR spectrum of **4**.

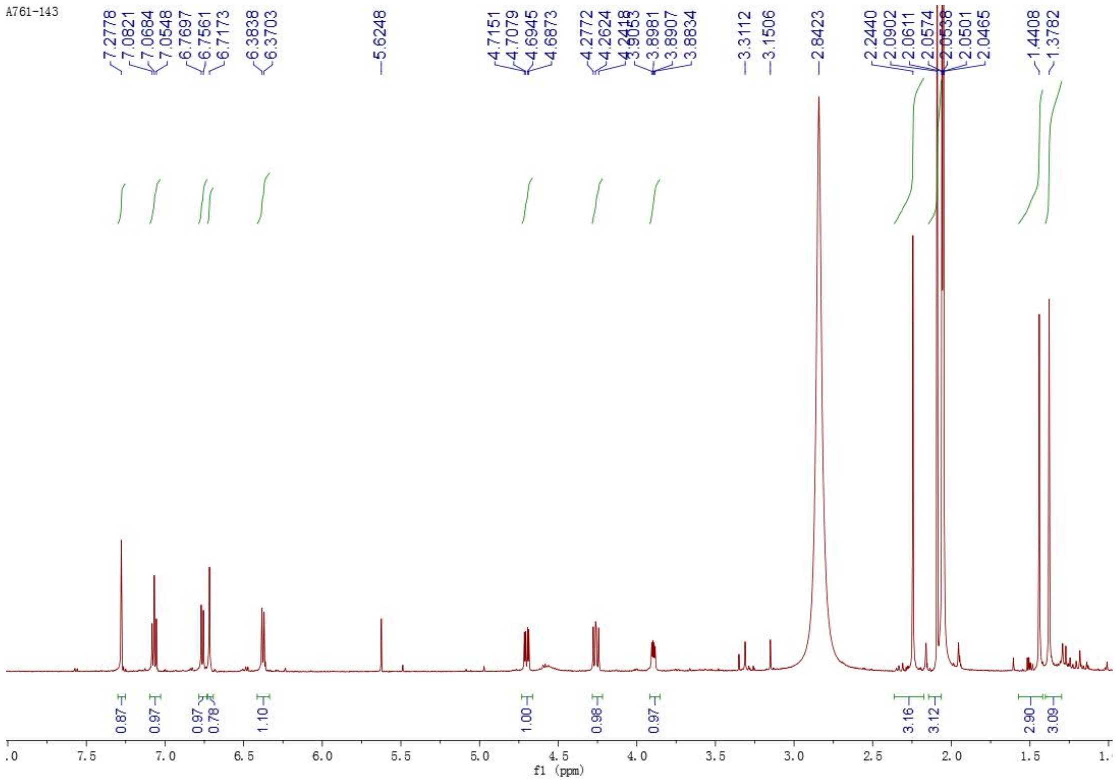

Figure S36. ^{1}H NMR spectrum (600 MHz, CD_3COCD_3) of **5**.

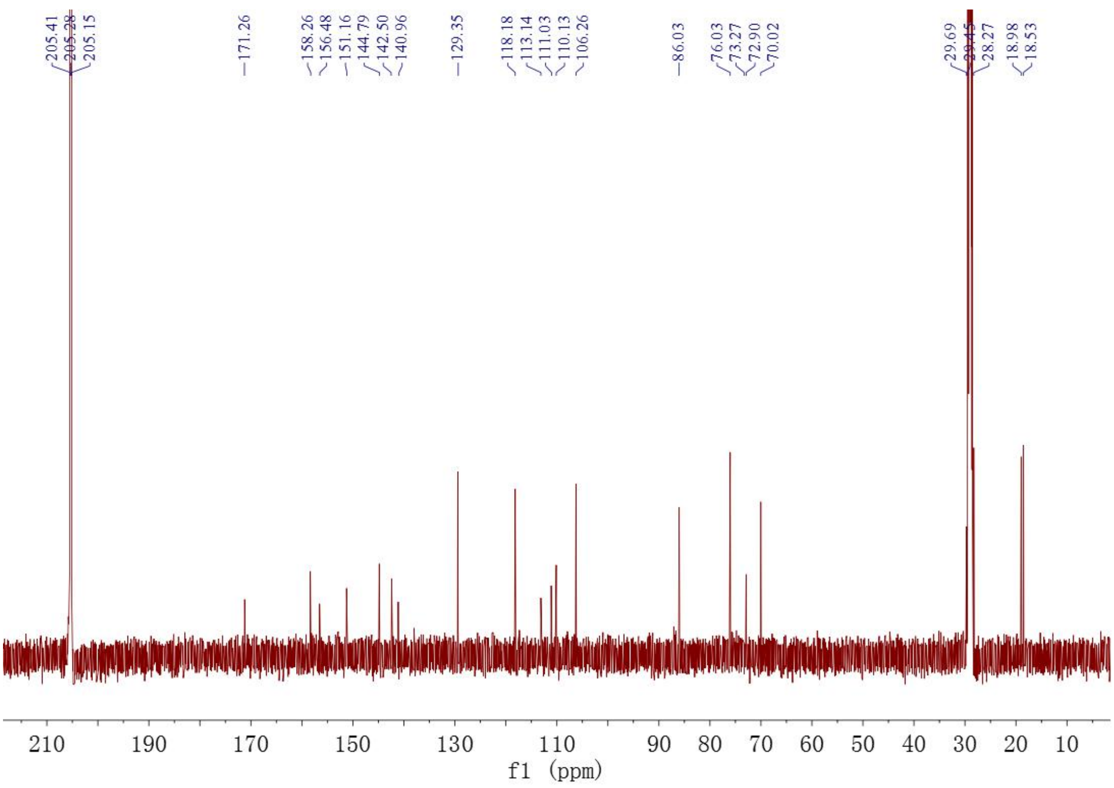

Figure S37. ^{13}C NMR spectrum (150 MHz, CD_3COCD_3) of **5**.

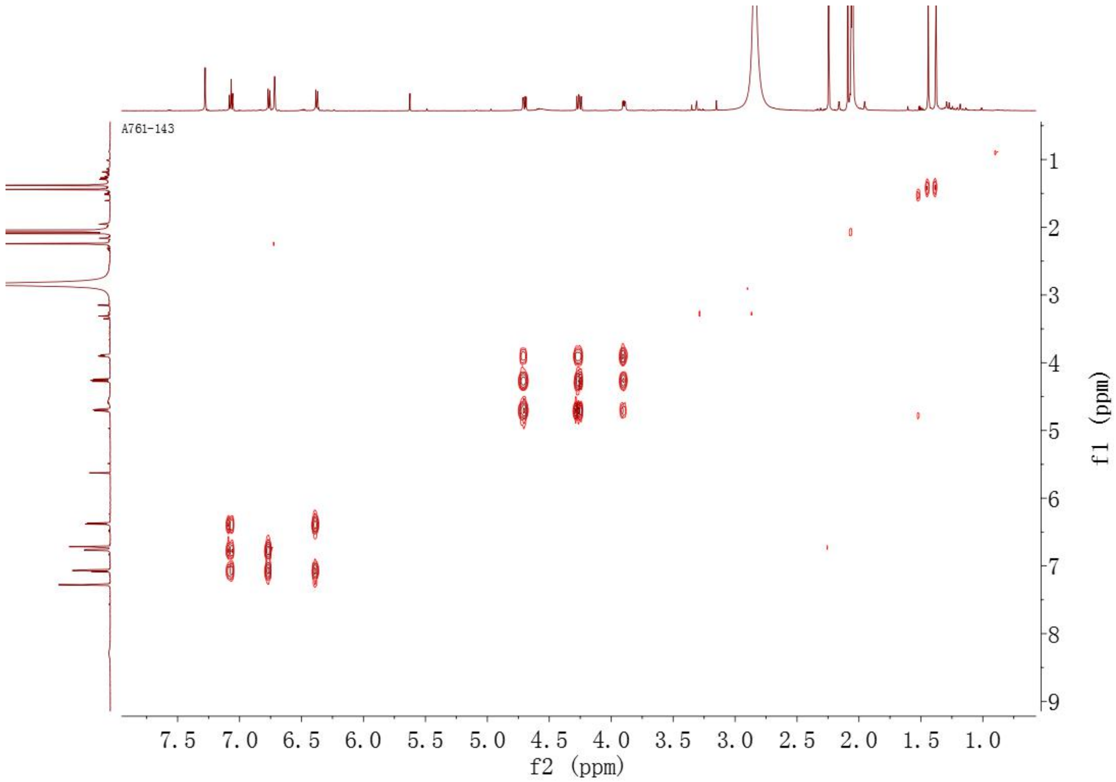

Figure S38. ^{1}H-^{1}H COSY spectrum (600 MHz, CD_3COCD_3) of **5**.

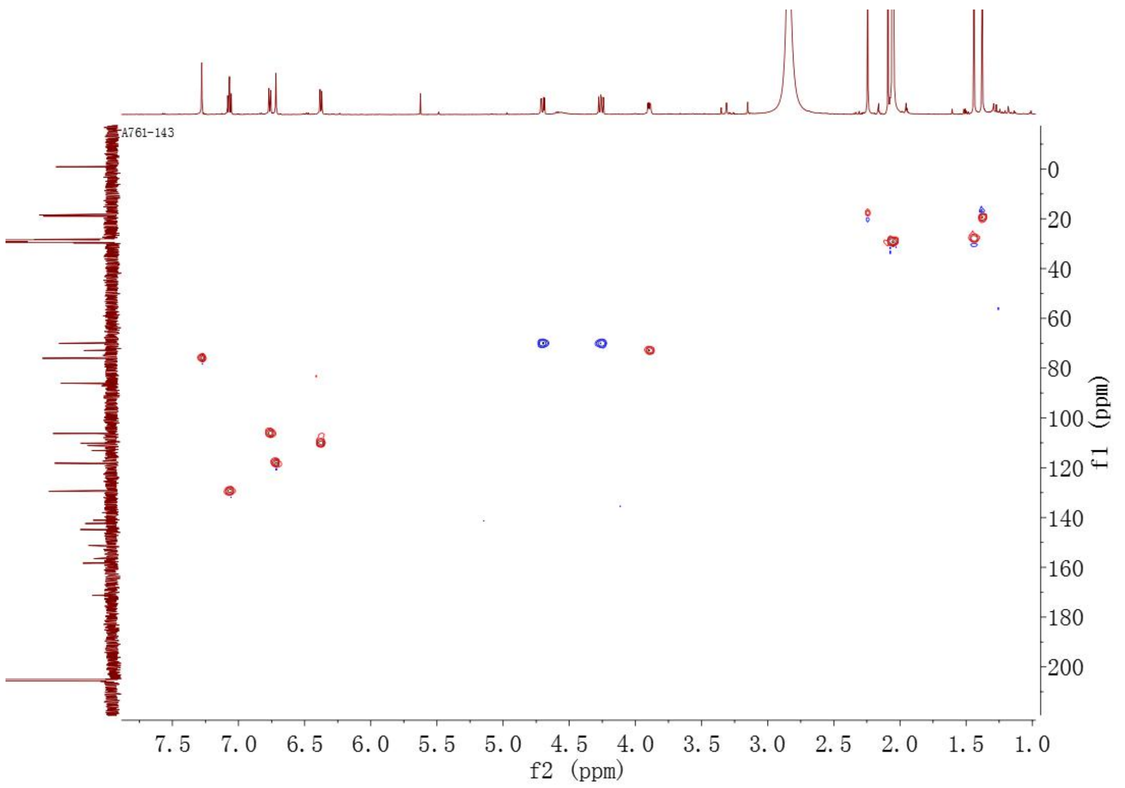

Figure S39. HSQC spectrum of **5**.

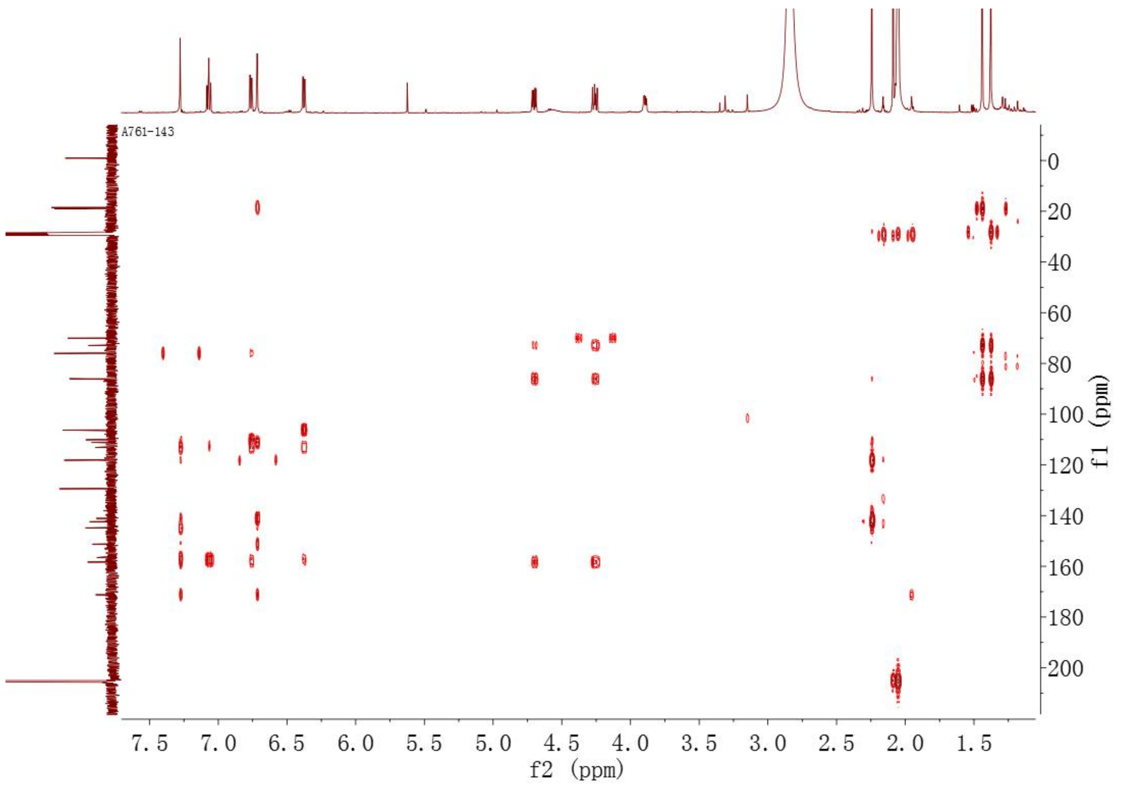

Figure S40. HMBC spectrum of **5**.

Mass Spectrum SmartFormula Report

Analysis Info

Analysis Name	D:\Data\MS\data\201812\liuhongxin_A761-143_pos_44_01_5937.d	Acquisition Date	12/13/2018 11:58:04 AM
Method	LC_Direct Infusion_pos_70-500mz.m	Operator	SCSIO
Sample Name	liuhongxin_A761-143_pos	Instrument	maXis 255552.00029
Comment			

Acquisition Parameter

Source Type	ESI	Ion Polarity	Positive	Set Nebulizer	0.4 Bar
Focus	Active	Set Capillary	4500 V	Set Dry Heater	180 °C
Scan Begin	70 m/z	Set End Plate Offset	-500 V	Set Dry Gas	4.0 l/min
Scan End	1500 m/z	Set Charging Voltage	0 V	Set Divert Valve	Waste
		Set Corona	0 nA	Set APCI Heater	0 °C

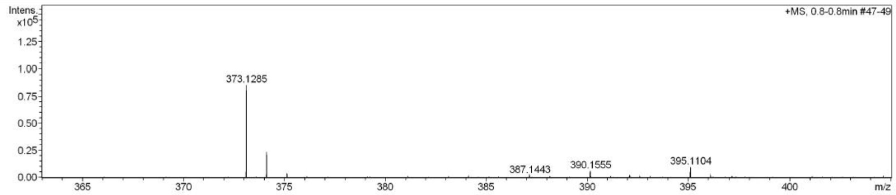

Meas. m/z	#	Ion Formula	Score	m/z	err [ppm]	err [mDa]	mSigma	rdb	e⁻ Conf	N-Rule
373.1285	1	C20H21O7	100.00	373.1282	-0.9	-0.3	28.6	10.5	even	ok
395.1104	1	C20H20NaO7	100.00	395.1101	-0.8	-0.3	30.5	10.5	even	ok
745.2493	1	C40H41O14	100.00	745.2491	0.3	0.2	35.6	20.5	even	ok
767.2312	1	C40H40NaO14	100.00	767.2310	0.3	0.2	7.8	20.5	even	ok

liuhongxin_A761-143_pos_44_01_5937.d
Bruker Compass DataAnalysis 4.1 printed: 12/13/2018 2:36:56 PM by: SCSIO Page 1 of 1

Figure S41. HRESIMS spectrum of **5**.

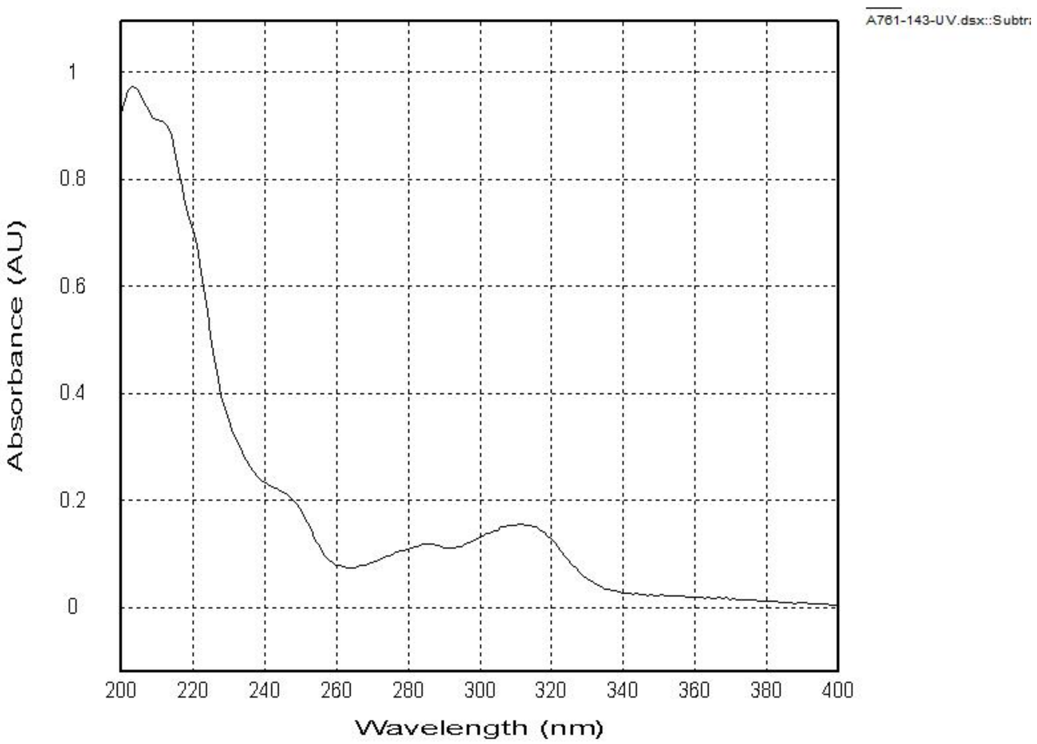

Figure S42. UV spectrum of **5**.

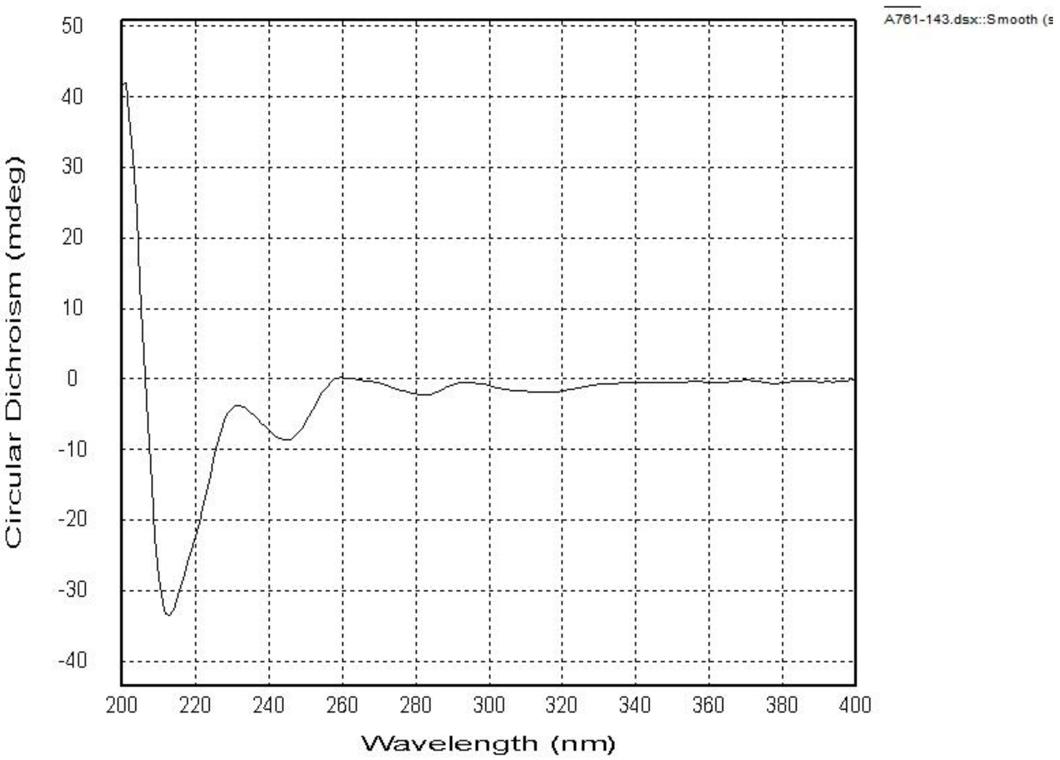

Figure S43. CD spectrum of **5**.

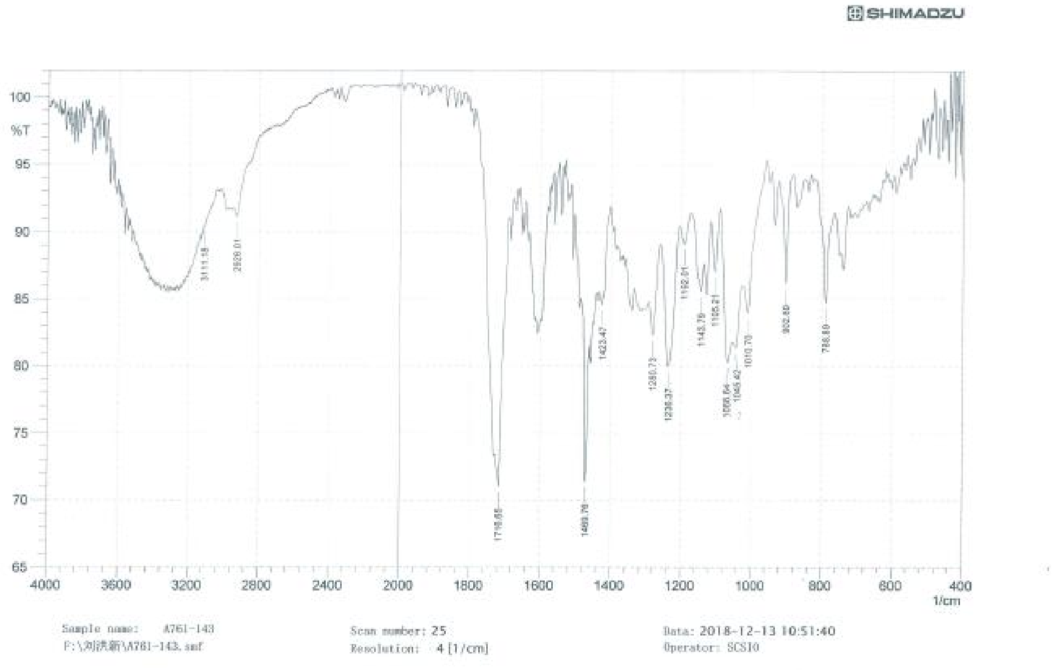

Figure S44. IR spectrum of **5**.

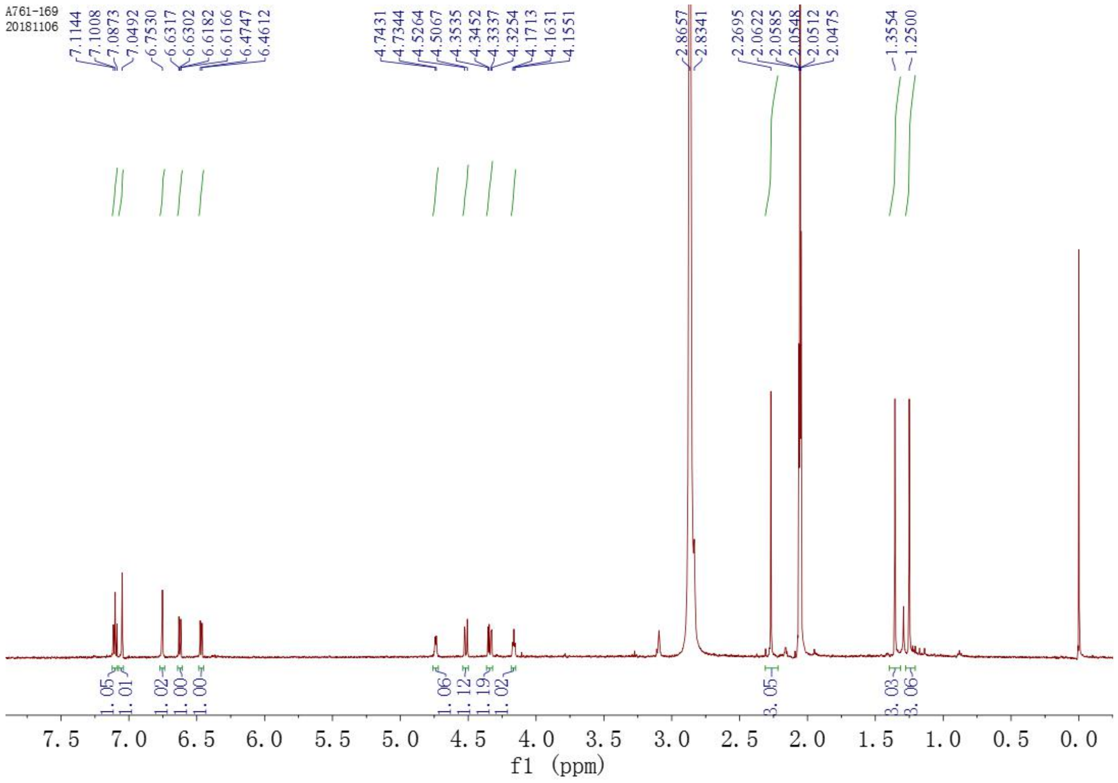

Figure S45. ^{1}H NMR spectrum (600 MHz, CD_3COCD_3) of **6**.

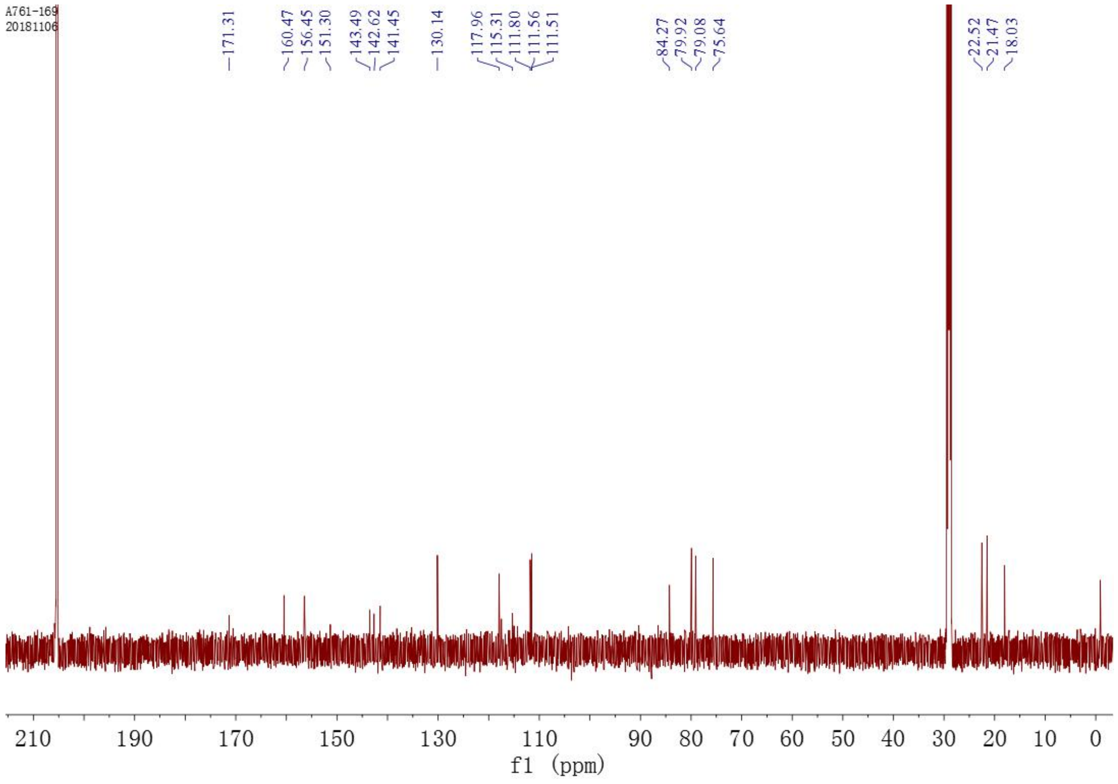

Figure S46. ^{13}C NMR spectrum (150 MHz, CD_3COCD_3) of **6**.

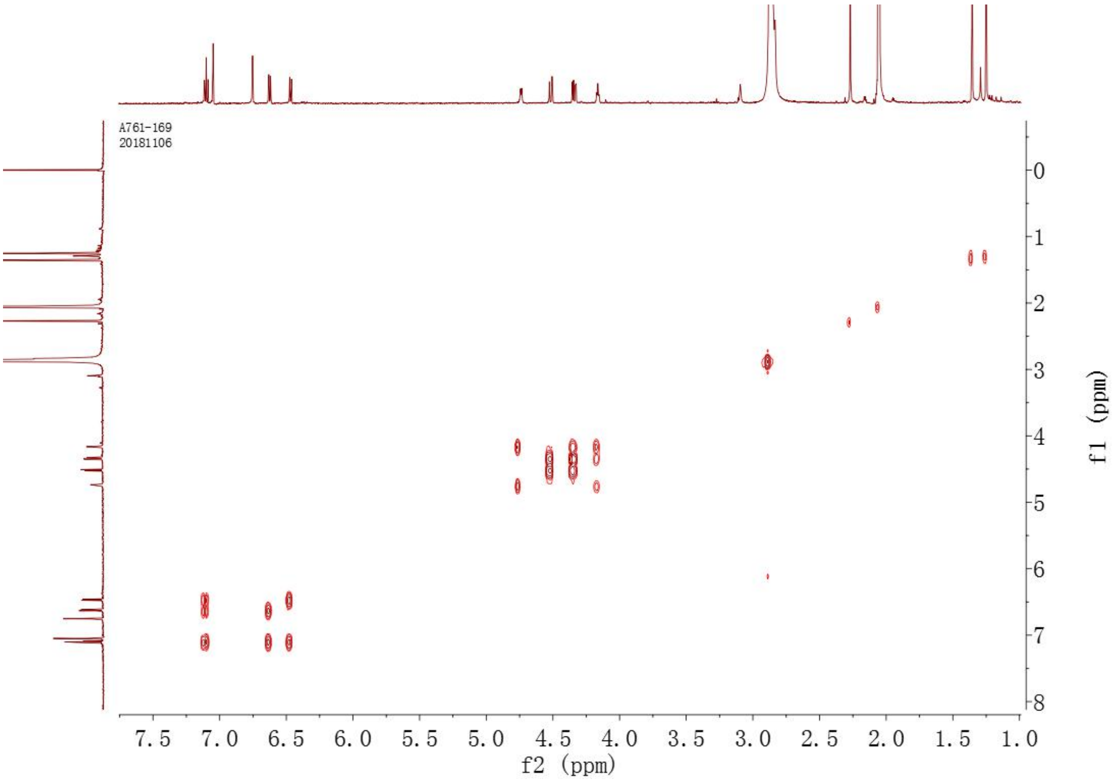

Figure S47. ^{1}H-^{1}H COSY spectrum (600 MHz, CD_3COCD_3) of **6**.

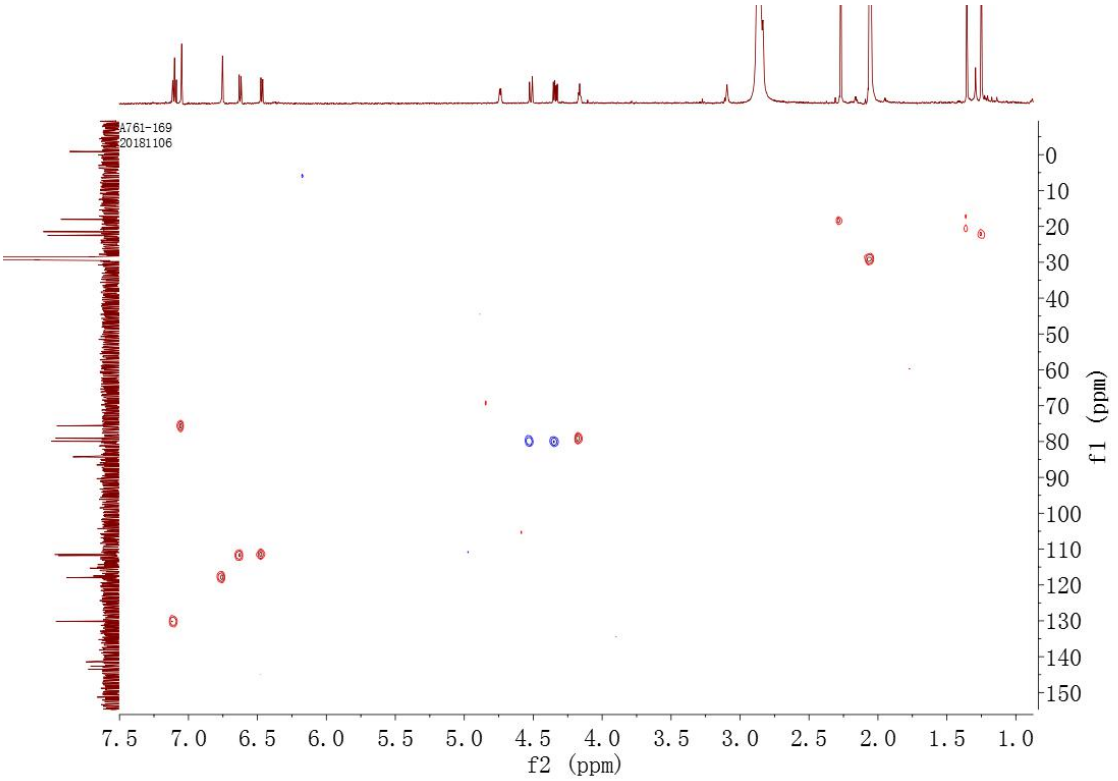

Figure S48. HSQC spectrum of **6**.

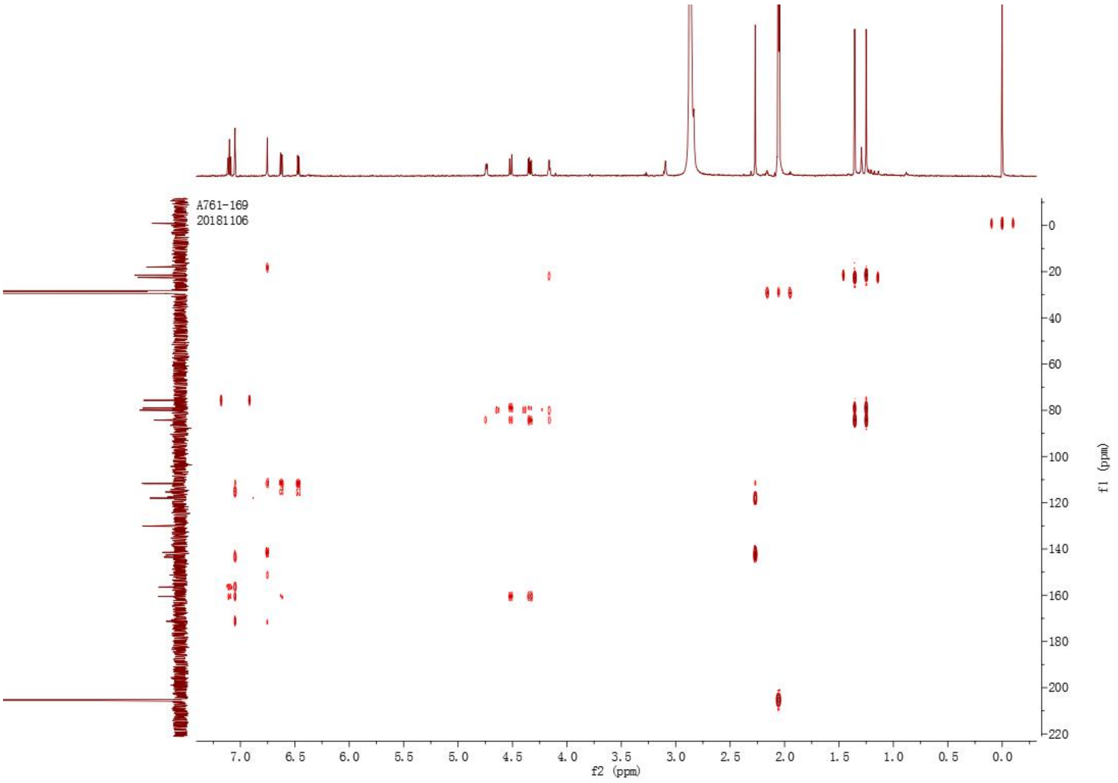

Figure S49. HMBC spectrum of **6**.

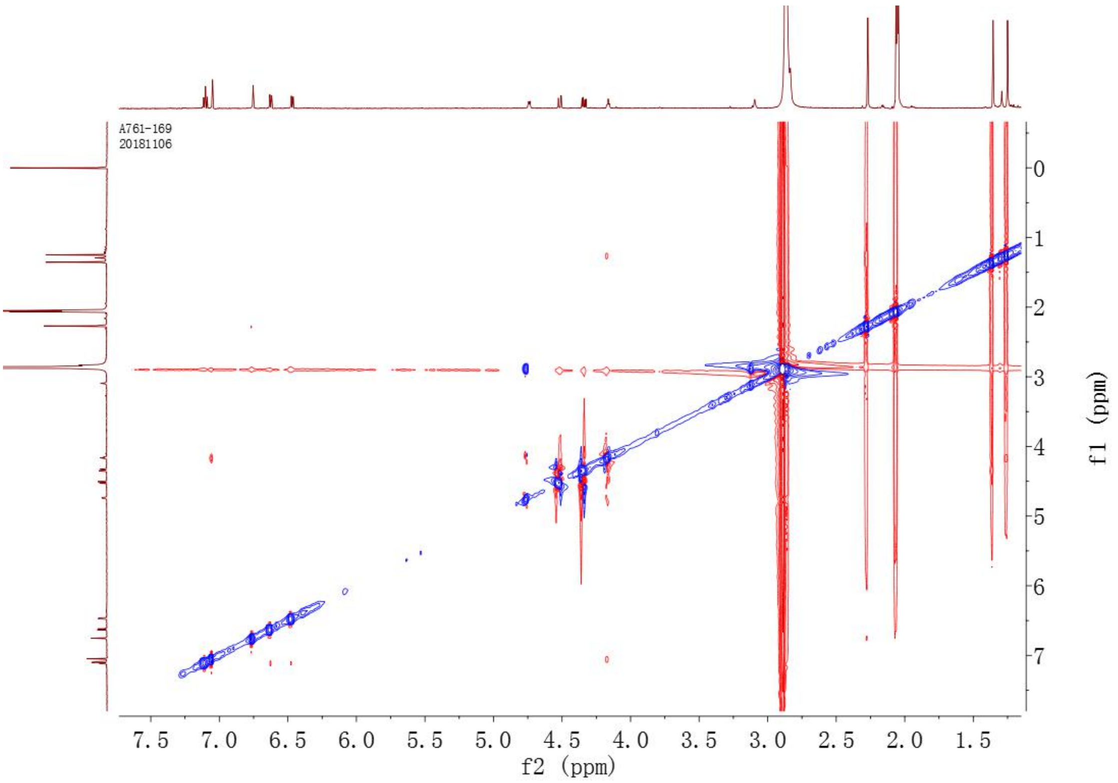

Figure S50. NOESY spectrum of **6**.

Mass Spectrum SmartFormula Report

Analysis Info

Analysis Name	D:\Data\MS\data\201812\liuhongxin_A761-169_pos_45_01_5938.d	Acquisition Date	12/13/2018 12:01:32 PM
Method	LC_Direct Infusion_pos_70-500mz.m	Operator	SCSIO
Sample Name	liuhongxin_A761-169_pos	Instrument	maXis 255552.00029
Comment			

Acquisition Parameter

Source Type	ESI	Ion Polarity	Positive	Set Nebulizer	0.4 Bar
Focus	Active	Set Capillary	4500 V	Set Dry Heater	180 °C
Scan Begin	70 m/z	Set End Plate Offset	-500 V	Set Dry Gas	4.0 l/min
Scan End	1500 m/z	Set Charging Voltage	0 V	Set Divert Valve	Waste
		Set Corona	0 nA	Set APCI Heater	0 °C

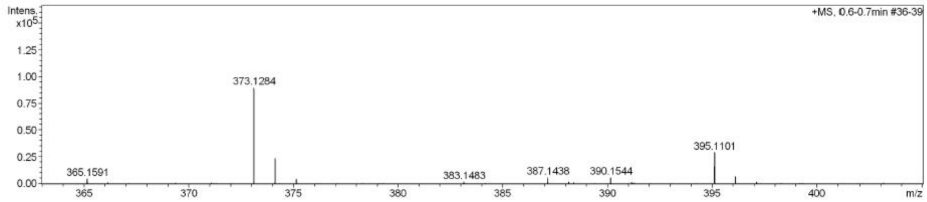

Meas. m/z	#	Ion Formula	Score	m/z	err [ppm]	err [mDa]	mSigma	rdb	e⁻ Conf	N-Rule
373.1284	1	$C_{20}H_{21}O_7$	100.00	373.1282	-0.7	-0.2	21.5	10.5	even	ok
395.1101	1	$C_{20}H_{20}NaO_7$	100.00	395.1101	0.1	0.0	11.0	10.5	even	ok
745.2489	1	$C_{40}H_{41}O_{14}$	100.00	745.2491	-0.2	-0.2	32.8	20.5	even	ok
767.2317	1	$C_{40}H_{40}NaO_{14}$	100.00	767.2310	-0.9	-0.7	7.2	20.5	even	ok

liuhongxin_A761-169_pos_45_01_5938.d
Bruker Compass DataAnalysis 4.1 printed: 12/13/2018 2:39:01 PM by: SCSIO Page 1 of 1

Figure S51. HRESIMS spectrum of **6**.

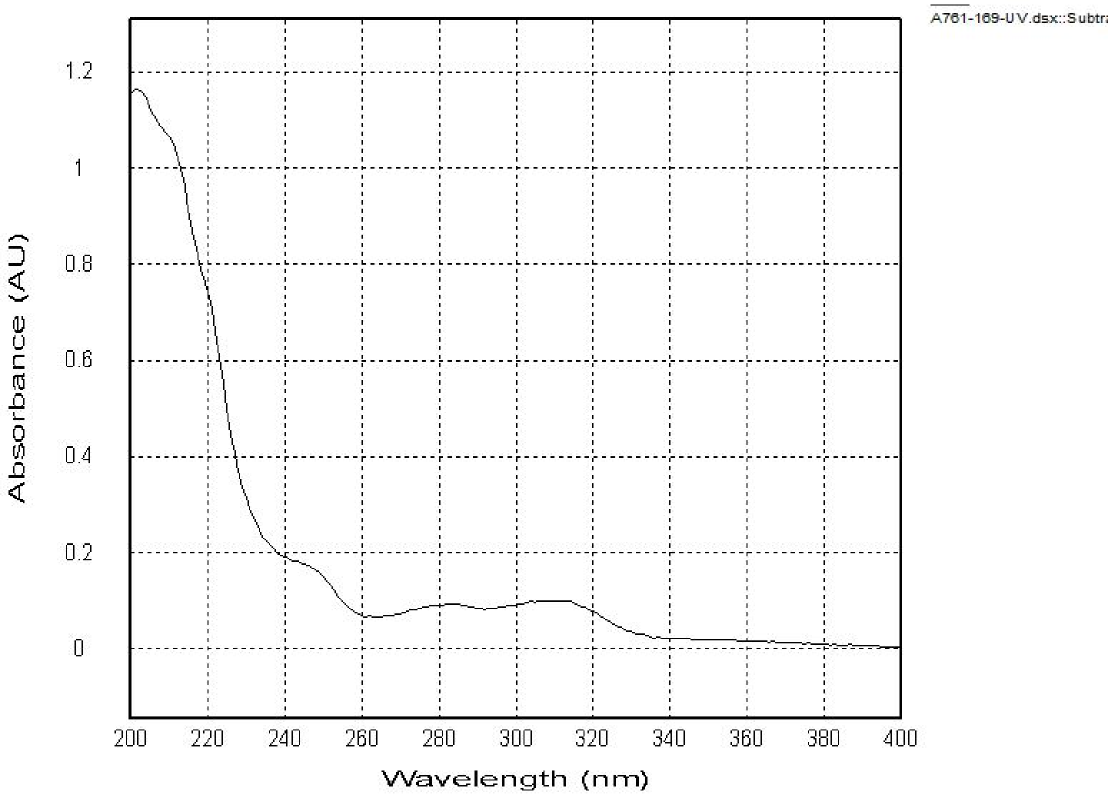

Figure S52. UV spectrum of **6**.

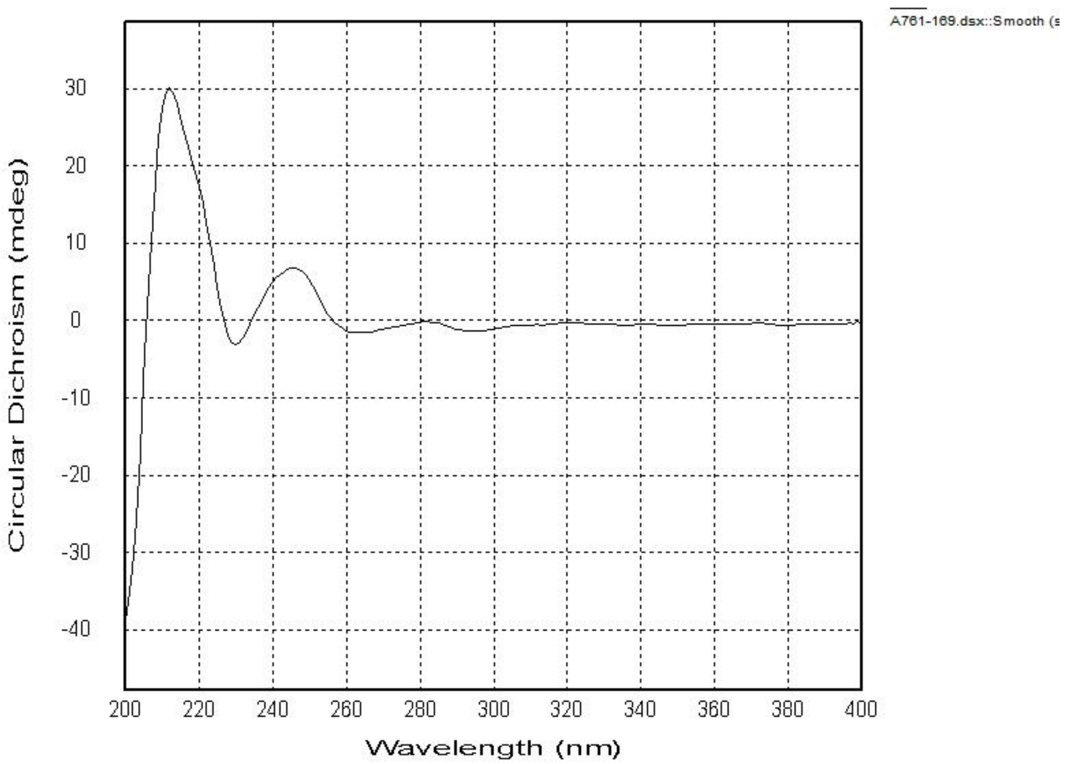

Figure S53. CD spectrum of **6**.

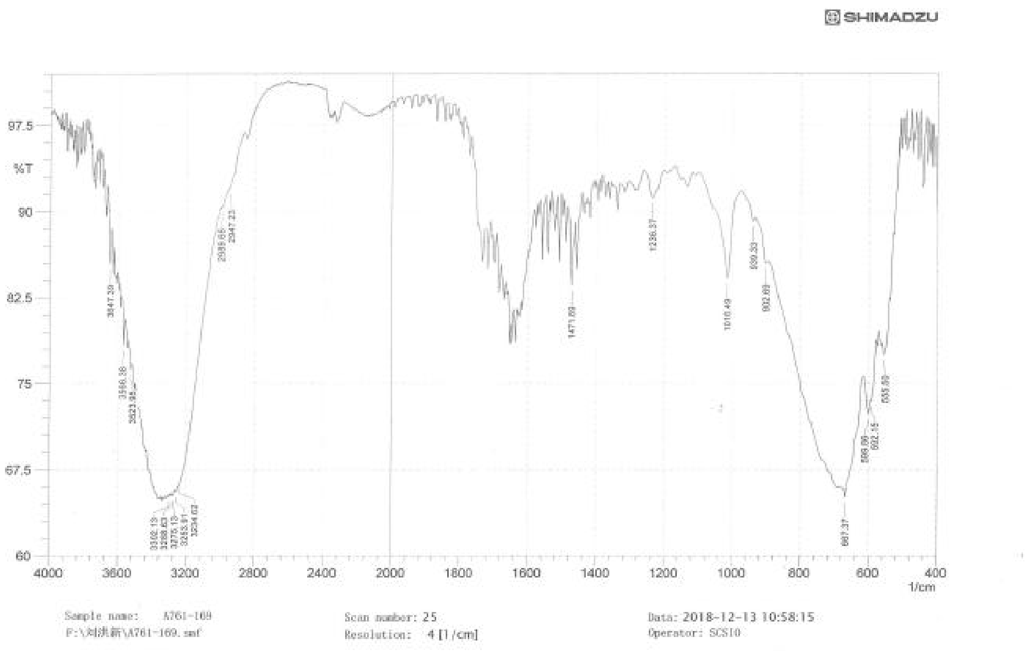

Figure S54. IR spectrum of **6**.
